# PRDM16 regulates smooth muscle cell identity and atherosclerotic plaque composition

**DOI:** 10.1038/s44161-025-00737-8

**Published:** 2025-10-17

**Authors:** Josephine M. E. Tan, Lan Cheng, Ryan P. Calhoun, Angela H. Weller, Karima Drareni, Skylar Fong, Eirlys Barbara, Hee-Woong Lim, Chenyi Xue, Hanna Winter, Gaëlle Auguste, Clint L. Miller, Muredach P. Reilly, Lars Maegdefessel, Esther Lutgens, Patrick Seale

**Affiliations:** 1 Institute for Diabetes, Obesity and Metabolism, University of Pennsylvania, Philadelphia, PA USA; 2 Department of Cell and Developmental Biology, Perelman School of Medicine, University of Pennsylvania, Philadelphia, PA USA; 3 Department of Physiology, Amsterdam Cardiovascular Sciences, Amsterdam University Medical Centers, Amsterdam, Netherlands; 4 Department of Biomedical Informatics, Cincinnati Children’s Hospital Medical Center, Cincinnati, OH USA; 5 Department of Pediatrics, University of Cincinnati, Cincinnati, OH USA; 6 Division of Cardiology, Department of Medicine, Columbia University Irving Medical Center, Irving Institute for Clinical and Translational Research, Columbia University, New York, NY USA; 7 Institute of Molecular Vascular Medicine, University Hospital rechts der Isar, Technical University Munich, Munich, Germany; 8 German Center for Cardiovascular Research (DZHK), partner site Munich Heart Alliance, Berlin, Germany; 9 Department of Genome Sciences, Department of Biochemistry and Molecular Genetics, University of Virginia, Charlottesville, VA USA; 10 Experimental Cardiovascular Immunology Laboratory, Department of Cardiovascular Medicine and Immunology, Mayo Clinic, Rochester, MN USA

**Keywords:** Atherosclerosis, Disease model, Atherosclerosis

## Abstract

Vascular smooth muscle cells (SMCs) undergo phenotype switching to acquire various fates in response to pathological stimuli. Among these, ‘synthetic’ SMCs—defined by migration, proliferation and extracellular matrix production—accumulate in atherosclerotic lesions and contribute to fibrous cap formation. The mechanisms driving this synthetic transition remain unclear. Here we identify *PRDM16*, a gene linked to cardiovascular disease, as a critical transcriptional repressor of the synthetic SMC phenotype. *PRDM16* expression declined during SMC modulation, and its deletion in mice induced a synthetic program across all SMC subtypes even without pathological stimuli. Under atherogenic conditions, *PRDM16* deficiency resulted in the formation of fibroproliferative plaques with more synthetic SMCs and fewer foam cells. Conversely, enforced PRDM16 expression suppressed SMC migration, proliferation and fibrosis. Mechanistically, PRDM16 occupied chromatin and suppressed activating marks at synthetic loci. These findings establish PRDM16 as a gatekeeper of SMC fate and reveal its role in shaping atherosclerotic plaque composition.

## Abstract (web summary)

Tan et al. identify PRDM16 as a key repressor of fibrotic switching in smooth muscle cells and show that its downregulation in atherosclerosis drives smooth muscle cells toward a synthetic fate, promoting fibrous plaques.

## Main

Cardiovascular diseases (CVDs) are a leading cause of mortality and morbidity worldwide. The primary underlying cause is atherosclerosis, a chronic inflammatory disease marked by the accumulation of lipid-rich plaques in artery walls^1,2^. Atherosclerotic lesion development begins with endothelial cell (EC) activation in response to dyslipidemia and other pathogenic stimuli, leading to monocyte adhesion, infiltration and differentiation into pro-inflammatory foam cells^3,4^. Within this pro-atherogenic environment, vascular smooth muscle cells (SMCs), which normally exist in a quiescent or contractile state, undergo ‘phenotypic switching’ or ‘modulation’, giving rise to various cell types. Among these, synthetic modulated SMCs migrate, proliferate and secrete extracellular matrix (ECM) components to help create a fibrous cap that surrounds lesions. SMCs can also give rise to ‘macrophage-like’ and ‘osteogenic’ cells in plaques^5–8^.

SMC phenotypic modulation is increasingly recognized as a major driver of atherosclerotic disease, with modulated SMCs comprising the majority of plaque cells (40–70%) and playing a central role in shaping plaque composition and clinical outcomes^6,9,10^. Plaque rupture can produce a thrombus and cause a potentially lethal cardiovascular event such as myocardial infarction or stroke. Autopsy studies indicate that ~70% of thrombi are caused by ruptured plaques, with ~80% of those plaques representing thin-capped fibroatheromas, composed of a large lipid or necrotic core with high immune cell infiltration, covered by a thin fibrous cap^11,12^. Thick fibrous caps rich in synthetic SMCs are thought to protect against rupture, whereas elevated immune cell activity and macrophage-like SMCs are associated with the development of more vulnerable plaques^13,14^. The other main cause of atherothrombosis is plaque erosion, characterized by endothelial loss, low inflammation and high synthetic SMC content^12,15^. Here, the role of synthetic SMCs is less explored, and it remains unclear whether their increased number is protective or deleterious. Given the clinical importance of SMC phenotypic modulation, defining the molecular mechanisms governing this process is a research priority.

Genome-wide association studies (GWAS), combined with single-nucleus chromatin accessibility profiling of patient arteries, recently nominated PR-domain containing 16 (*PRDM16*) as a candidate driver gene for coronary artery disease^16^. PRDM16 is an epigenetic and transcriptional regulator that has been extensively studied in adipocytes, where it drives an energy-burning metabolic program^17–19^. PRDM16 also regulates mitochondrial and metabolic programs in other cell types, including cardiomyocytes, intestinal epithelial cells and hematopoietic stem cells^20–23^, and has emerging roles in regulating ventricular cardiomyocyte and arteriovenous EC function^24–26^.

In this study, we investigated the role of PRDM16 in regulating SMC identity and atherosclerosis. We found that *PRDM16* is highly expressed in arterial SMCs and downregulated during SMC modulation in mouse and human atherosclerosis. Acute or chronic loss of *Prdm16* in SMCs of mice activated a synthetic gene program in SMCs even under homeostatic (basal) conditions. Upon atherogenic challenge, mice with SMC-selective loss of *Prdm16* developed fibrous, collagen-rich plaques, characterized by enhanced proliferation of synthetic SMCs and reduced foam cell content. Single-cell RNA sequencing (scRNA-seq) analyses revealed that loss of *Prdm16* upregulated synthetic genes across all SMC subpopulations and promoted the emergence of synthetic modulated cells during atherosclerosis. Conversely, enforced PRDM16 expression in vitro potently repressed synthetic processes, including migration, proliferation and fibrosis through chromatin binding at target loci. Overall, these results define PRDM16 as a critical repressor of the synthetic phenotype in SMCs and as a determinant of atherosclerotic plaque composition.

## Results

### *PRDM16* is a CVD-associated gene with enriched expression in SMCs

**Fig. 1:**
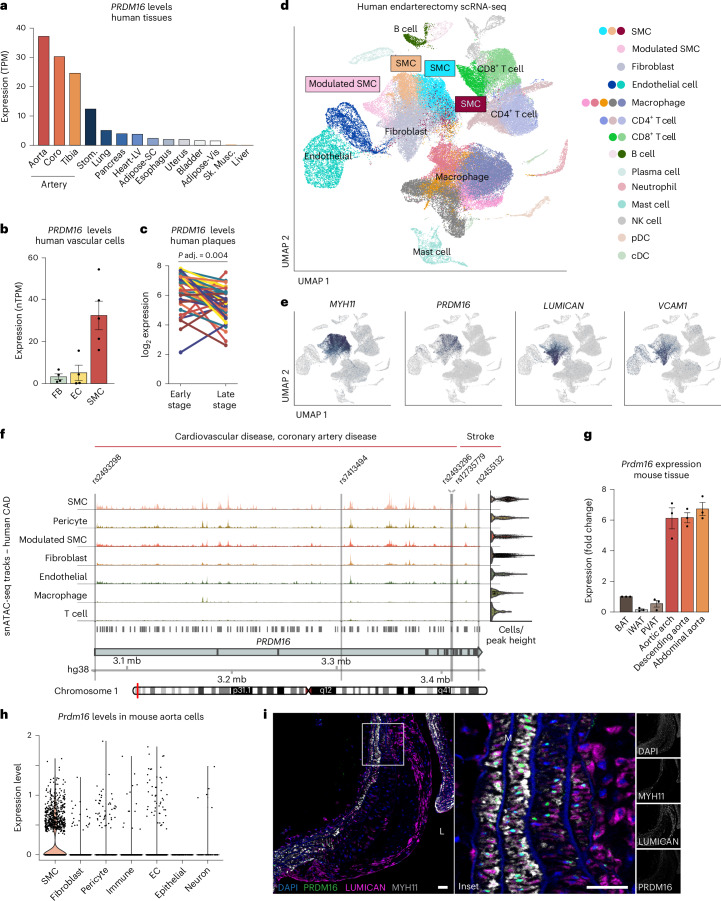
*PRDM16* expression decreases during SMC modulation in mouse and human atherosclerosis. **a**, *PRDM16* expression in human tissues from the GTEx database in transcripts per million (TPM) (dbGaP accession phs000424.v8.p2). Coro, coronary; Stom, stomach; LV, left ventricular; Vis, visceral; Sk. Musc.; skeletal muscle. **b**, *PRDM16* mRNA levels in vascular cells in number of transcripts per million (nTPM), *n* = 3 (Human Protein Atlas). Values represent mean ± s.e.m. **c**, Expression of *PRDM16* in endarterectomy samples of paired early- and late-stage lesions from the same human patients^26^, *n* = 38. A paired *t*-test was performed, and the adjusted *P* value (*P* adj.) is shown after false discovery rate correction for multiple testing. **d**, UMAP visualization of clusters identified by scRNA-seq of human endarterectomy samples^27^, *n* = 21. NK, natural killer; pDC, plasmacytoid dendritic cell; cDC, conventional dendritic cell. **e**, UMAP feature plots showing expression of *PRDM16*, *MYH11* and modulated SMC marker genes *LUMICAN* and *VCAM1*. **f**, Visualization of snATACseq tracks at the *PRDM16* locus from human coronary artery samples (*N* = 41 patients)^15^, clustered by cell type and showing the locations of SNPs associated with CVD and/or stroke. m.b., megabase. **g**, *Prdm16* mRNA levels in mouse BAT, inguinal WAT (iWAT), PVAT, aortic arch, descending aorta and abdominal aorta. Values represent mean ± s.e.m, *n* = 3 mice. **h**, Violin plot showing *Prdm16* mRNA levels (TPM) in cell clusters from mouse aortas^29^. **i**, Immunostaining for PRDM16 (green), MYH11 (white), Lumican (magenta) and DAPI (nuclei, blue) in sagittal section of aorta containing a lesion from mice treated with AAV8-PCSK9-D377Y and western diet for 12 weeks. Scale bar, 50 µm. M, tunica media, L, lumen. Representative of *n* = 6 mice. Source data

GWAS have identified several single-nucleotide polymorphisms (SNPs) within the *PRDM16* locus that are associated with increased CVD risk (Extended Data Table 1). In addition, rare *PRDM16* coding variants in the UK Biobank cohort are associated with atherosclerosis-associated conditions, including coronary artery disease, stroke and CVD. Analysis of *PRDM16* mRNA expression across human tissues using the Genotype-Tissue Expression (GTEx) portal revealed high expression in arterial tissues, including aorta, coronary arteries and tibial arteries, compared with other organs (Fig. 1a). Single-cell transcriptomic analysis of human arteries showed a striking >10-fold enrichment of *PRDM16* mRNA in SMCs relative to ECs and fibroblasts (Fig. 1b).

### PRDM16 is downregulated during SMC phenotypic modulation in atherosclerosis

**Extended Data Fig. 1:**
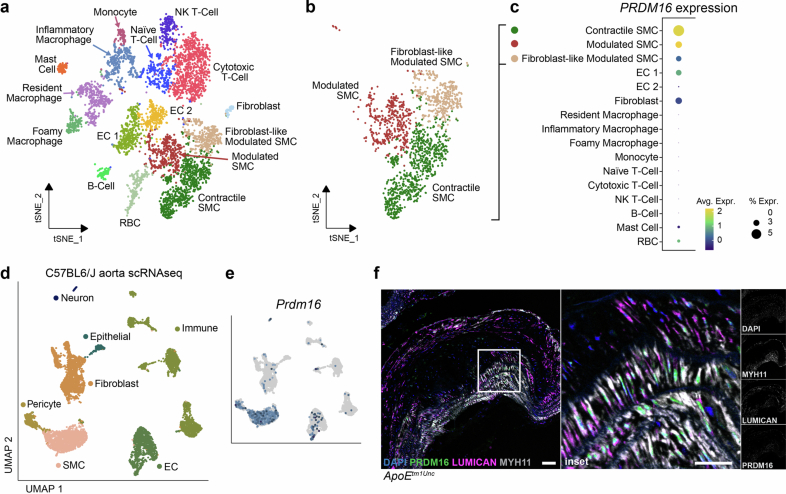
PRDM16 is expressed in SMCs and downregulated during atherosclerosis. **a**. tSNE showing cell clusters from scRNAseq of human carotid artery plaques, n=9. **b**. SMC clusters from (**b**). **c**. Dot plot showing *PRDM16* expression in all clusters from (**b**). **d**. UMAP of gene expression in 6,753 cells from adult thoracic aortae of 13-week-old male CD1 mice29. **e**. UMAP feature plot of *Prdm16* expression in clusters from (**e**). **f**. Immunostaining for PRDM16 (green), MYH11 (white), Lumican (magenta) and DAPI (nuclei, blue) in a sagittal section of an aortic lesion from *Apoe-/-* mice fed a western diet for 18 weeks. Scale bar: 50 µm. Representative of n=3 mice.

Phenotypic modulation of SMCs during human atherosclerosis is characterized by the downregulation of contractile SMC marker genes such as *MYH11*, *TAGLN* and *CNN1*, and activation of gene programs that promote vascular remodeling, lesion development and fibrous cap formation. Analysis of paired early- and late-stage human atherosclerotic lesions revealed significantly lower *PRDM16* expression in late-stage plaques, which are enriched in modulated SMCs^27^ (Fig. 1c). Single-cell transcriptomic profiling of human plaques^28^ showed that *PRDM16* was highly expressed in canonical SMCs (*MYH11⁺*, *TAGLN⁺*) and markedly decreased in modulated SMCs expressing *LUM* and *COL1A1* (Fig. 1d,e). Analysis of a second scRNA-seq dataset from human carotid plaques^29^ corroborated these findings, showing robust *PRDM16* expression in contractile SMCs and greatly diminished levels in modulated and fibroblast-like SMC subpopulations (Extended Data Fig. 1a–c). Single-nucleus assay for transposase-accessible chromatin using sequencing (ATAC-seq) analysis of coronary artery disease patient samples^16^ further demonstrated that chromatin accessibility at the *PRDM16* locus was highest in SMCs and lower in pericytes and modulated SMCs (Fig. 1f). Notably, several coronary artery disease-associated SNPs mapped to accessible chromatin regions within the *PRDM16* locus in SMCs (Fig. 1f and Extended Data Table 1).

*Prdm16* exhibited a similar expression pattern in mice, with higher levels in all aortic regions compared with tissues where *Prdm16* has been most studied, such as brown adipose tissue (BAT), perivascular adipose tissue (PVAT) and subcutaneous white adipose tissue (WAT) (Fig. 1g). Single-cell analysis of mouse aortae^30^ showed that *Prdm16* expression was highly enriched in SMCs relative to other vascular cell types (Fig. 1h and Extended Data Fig. 1d,e). We then assessed PRDM16 expression in mouse atherosclerosis. Immunofluorescence (IF) staining of lesions from two different mouse models of atherosclerosis showed that modulated SMCs, marked by Lumican expression, were concentrated in the fibrous cap, spatially separated from MYH11^+^ SMCs in the media (Fig. 1i and Extended Data Fig. 1f). PRDM16 protein was highly expressed in the nuclei of medial MYH11^+^ cells and was absent in LUM^+^ cells within the lesion. Intriguingly, PRDM16 expression declined across the lesion axis, from high levels in medial SMCs to undetectable levels in distal cap regions (Fig. 1i and Extended Data Fig. 1f). Collectively, these data demonstrate that PRDM16 is highly expressed in arterial SMCs and downregulated during SMC modulation and fibrotic cap formation in both human and mouse atherosclerosis.

### PRDM16 represses the synthetic gene program in SMCs under homeostatic conditions

**Extended Data Fig. 2:**
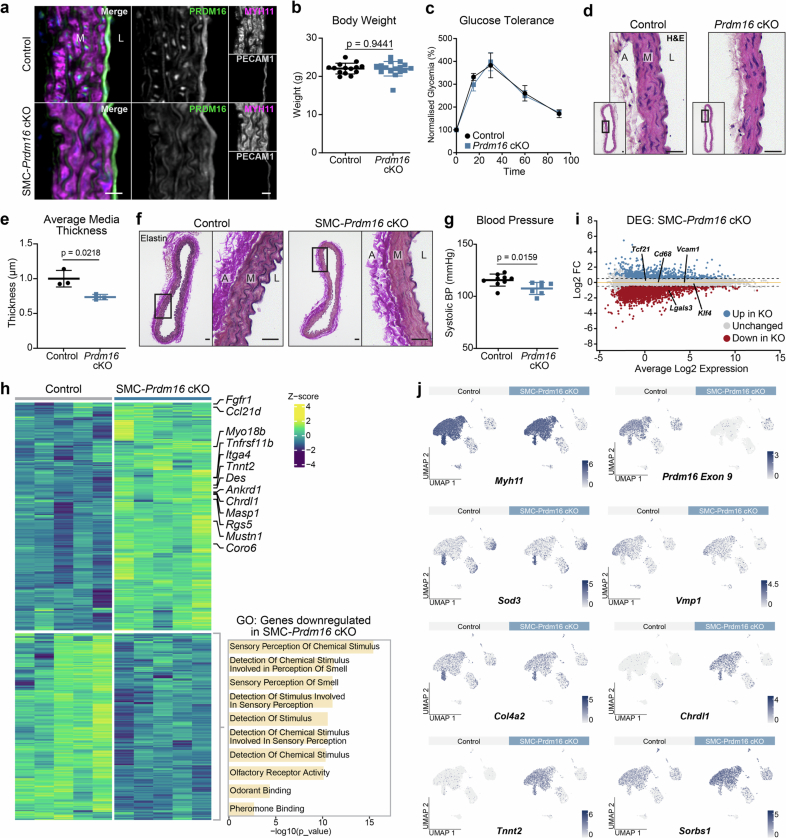
Loss of *Prdm16* in SMCs leads to upregulation of the synthetic gene program. **a**. Immunostaining for PRDM16 (green), MYH11 (magenta), PECAM1 (CD31, white), and DAPI (nuclei, blue) in descending aorta from control and cKO mice. Scale bar: 20 µm. **b**. Bodyweights of 8-week-old male control (n=14) and cKO (n=16) mice. Values represent mean ± S.D. **c**. Oral glucose tolerance test in mice from (b). Values represent mean ± S.E.M. (n=7 per group). **d**. H&E staining of descending aorta from control and cKO mice. Scale bar: 100 µm. **e**. Media thickness of descending aorta in control and cKO mice (average of 6 equally spaced-out measurements per aorta). Values represent mean ± S.D. (n=3 mice per group). An unpaired, two-tailed t-test was performed, with an F-test to compare variances (ns). **f**. Van Gieson stain for Elastin. Scale bar: 100 µm. **g**. Blood pressure (BP) in 10-week-old control (n=9) and cKO (n=7) mice (each dot = average BP from 5 days consecutive measurements). Data are represented as mean ± S.D. An unpaired, two-tailed t-test was performed. F-test for variance (ns) **h**. Heatmap of the top regulated genes in control vs. cKO aortae (n=5 per group). (bottom right) GO analyses of genes downregulated in cKO vs. control aortae. **i**. Expression MA plot (Fig. 2a), with unchanged SMC phenotypic transition genes annotated. **j**. UMAP visualization of indicated genes in control and cKO aortae.

To investigate the role of PRDM16 in SMCs, we generated mice with constitutive, SMC-selective deletion of *Prdm16* using the *Tagln*-Cre driver (SMC*-*cKO). IF analysis confirmed efficient ablation of PRDM16 in SMCs, while expression in ECs remained intact (Extended Data Fig. 2a). SMC-cKO mice were born at expected Mendelian ratios, appeared phenotypically normal and maintained body weight and glycemic control comparable to littermate controls (Extended Data Fig. 2b,c). Histological examination of aortic sections showed a ~30% reduction in media thickness, and decreased Elastin staining in SMC-cKO mice (Extended Data Fig. 2d–f). Systolic blood pressure was slightly lower in SMC-cKO relative to control mice but remained within the normal range (90–120 mm Hg) (Extended Data Fig. 2g).

**Fig. 2:**
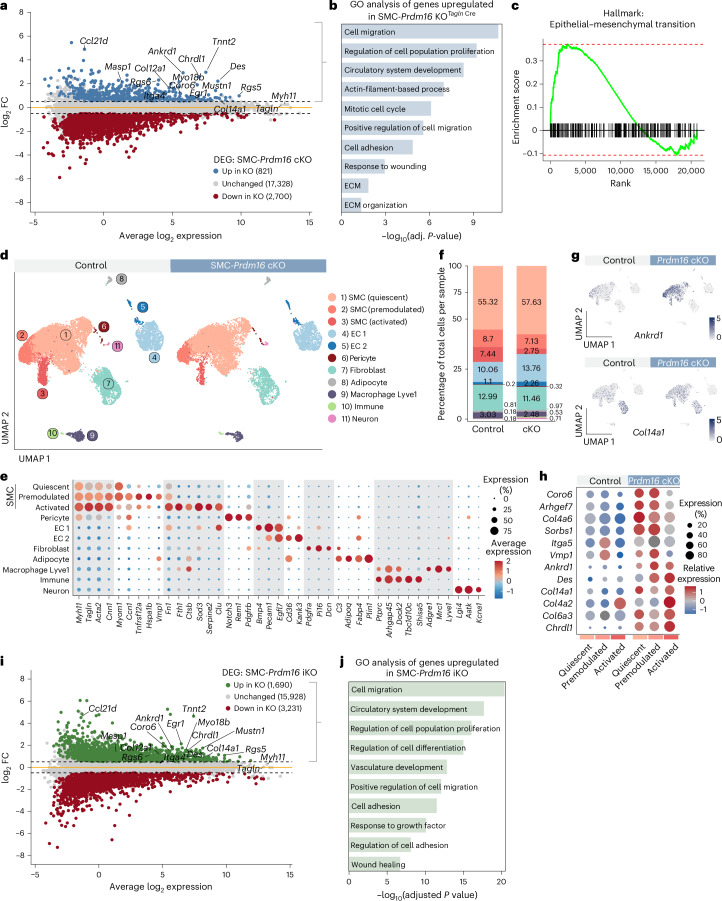
PRDM16 represses the synthetic gene program in SMCs under homeostatic conditions. **a**, Expression MA plot of differentially expressed genes between control and cKO aortae (*n* = 5 per group). Blue: genes upregulated in cKO (log_2_FC > 0.5 and *P* adj. <0.1). Red: genes downregulated in cKO (log_2_FC <−0.5 and *P* adj. <0.1). Gray: unchanged genes. **b**, GO analysis of genes upregulated cKO versus control aortae. **c**, Gene set enrichment analysis showing enrichment of hallmark process ‘epithelial to mesenchymal transition’ in cKO versus control aortae. **d**, UMAP visualization of scRNA-seq expression data from control (*n* = 4, 11,297 cells) and cKO aortae (*n* = 4, 9,118 cells). **e**, Dot plot showing the expression of cluster-defining genes. **f**, Bar charts showing the proportion of cells assigned to each cluster. **g**, UMAP feature plot of *Ankrd1* and *Col14a1* expression in clusters from **d**. **h**, Dot plot displaying synthetic gene levels in SMC clusters from control and cKO aortae. **i**, Expression MA plot of differentially expressed genes between control and iKO aortae (*n* = 3 per group). Green: genes upregulated in iKO (log_2_FC > 0.5 and *P* adj. <0.1). Red: genes downregulated in iKO (log_2_FC < −0.5 and *P* adj. <0.1). Gray: unchanged genes. **j**, GO analyses of genes upregulated in iKO versus control aortae. Source data

To determine the impact of *Prdm16* deletion on SMC gene expression, we performed bulk RNA-seq of cleaned aortae (stripped of perivascular tissue and endothelium), from SMC-cKO and control mice. Canonical SMC marker genes such as *Myh11* and *Tagln* were among the most abundantly expressed genes and showed no significant changes between genotypes (Fig. 2a). By contrast, many genes related to myofibroblast transition, fibrosis and ECM remodeling (*Ankrd1*, *Col12a1*, *Mustn1*, *Chrdl1*, *Des*, *Coro6* and *Egr1*) were upregulated in SMC-cKO aortae (Fig. 2a and Extended Data Fig. 2h). Gene Ontology (GO) analysis of the upregulated genes in cKO aortae revealed enrichment of pathways related to synthetic SMC modulation, including ‘cell migration’, ‘regulation of cell population proliferation’, ‘response to wounding’ and ‘extracellular matrix organization’ (Fig. 2b). Gene set enrichment analysis further identified ‘epithelial to mesenchymal transition’, a process related to synthetic SMC switching, as a top hallmark pathway (Fig. 2c). SMC-cKO aortae also expressed elevated levels of genes involved in cardiac/circulatory system development and markers of secondary heart field-derived SMCs (*Tnnt2* and *Myo18b*) (Fig. 2a,b). Downregulated genes mapped to nonspecific transcriptional and sensory/perception processes (Extended Data Fig. 2h). Notably, *Prdm16* deficiency did not alter the expression of *Tcf21* or *Klf4*, encoding key transcriptional regulators of SMC modulation^6,24^, nor genes associated with the macrophage-like SMC phenotype such as *Lgals3*, *Cd68* and *Vcam1*) (Extended Data Fig. 2i).

To obtain cellular resolution of transcriptional changes, we performed scRNA-seq using the 10x Genomics Flex platform on fixed aortae from control and SMC-cKO mice. A key advantage of this technique is that it preserves native transcriptional profiles before cell dissociation. Unsupervised clustering of 20,415 cells identified seven major clusters (and four minor clusters with <1% of cells per condition), including three SMC populations, two EC populations, adventitial fibroblasts and a macrophage cluster expressing the vascular-resident macrophage marker *Lyve1* (Fig. 2d–f). The largest SMC cluster corresponded to ‘quiescent’ SMCs, marked by expression of *Myh11*, *Tagln*, *Cnn1* and *Myom1*. A second cluster, termed ‘premodulated’ SMCs, expressed canonical SMC markers along with elevated levels of synthetic genes (*Ccn1*, *Tnfrsf12a*, *Vmp1* and *Fn1*). The third cluster, ‘activated’ SMCs, expressed contractile (*Myh11* and *Cnn1*), synthetic (*Fn1* and *Col4a2*) and ECM remodeling genes (*Ctsb*, *Clu* and *Sod3*) (Fig. 2e and Extended Data Fig. 2j). Using a custom probe targeting exon 9 (the floxed exon), we confirmed the efficient deletion of *Prdm16* in all SMC clusters (Extended Data Fig. 2j). Strikingly, the levels of synthetic marker genes (*Ankrd1*, *Col14a1*, *Col4a6*, *Coro6* and *Chrdl1*) were broadly and significantly elevated across all SMC subtypes in SMC-cKO compared with control mice (Fig. 2g,h and Extended Data Fig. 2j). Although premodulated and activated SMCs from control mice expressed detectable levels of certain synthetic marker genes, these genes were further upregulated by *Prdm16* deficiency (Fig. 2g,h and Extended Data Fig. 2j). Thus, the loss of *Prdm16* promotes the expression of synthetic genes in all types of SMCs under homeostatic conditions.

**Extended Data Fig. 3:**
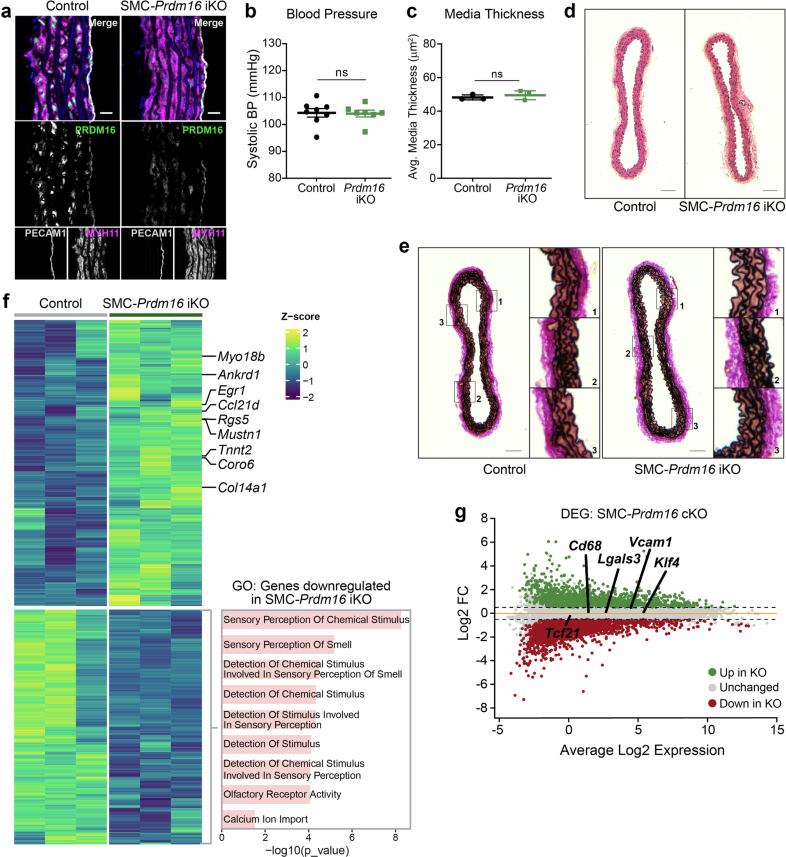
Acute loss of *Prdm16* in SMCs of adult mice leads to upregulation of the synthetic gene program. **a**. Immunostaining for MYH11 (magenta), PRDM16 (green), PECAM-1 (CD31, white), and DAPI (nuclei, blue) in descending aorta from control and iKO mice. Scale bar: 20 µm. **b**. Blood pressure in 10-week-old control (n=8) and iKO (n=7) mice. Values represent mean ± S.E.M. Each dot corresponds to average blood pressure of 4 days consecutive measurements. An unpaired, two-tailed t-test was performed, with F-test for variance (ns). **c**. Media thickness of descending aorta in control and iKO mice. Values represent mean ± S.D, and represent average of 6 equally spaced-out measurements per aorta (n=3 mice per group). An unpaired, two-tailed t-test was performed, with an F-test for variance (ns). **d**. H&E staining of descending aortae from control and iKO mice. Scale bar: 200 µm. **e**. Van Gieson stain for Elastin in control and iKO aortae. Scale bar: 200 µm. Representative of n=3 mice. **f**. Heatmap of top regulated genes in control vs. iKO aortae (n=5 per group). Bottom right: GO analysis of genes downregulated in iKO vs control aortae. **g**. Expression MA plot from Fig. 2i, with unchanged SMC phenotypic transition genes annotated. (A: tunica adventitia, M: tunica media, L: lumen). Source Data

To evaluate the effects of acute *Prdm16* deletion in SMCs of adult mice, we generated a tamoxifen-inducible *Myh11-CreERT*-driven *Prdm16* KO model (SMC-iKO). 8-week-old SMC-iKO and control mice were treated with tamoxifen to delete *Prdm16* specifically in SMCs. IF staining of aortae one-week post-treatment showed a near-complete loss of PRDM16 protein expression in SMCs of iKO mice (Extended Data Fig. 3a). Blood pressure, media thickness and elastic lamina structure were unchanged between control and iKO mice (Extended Data Fig. 3b–e). RNA-seq analysis of cleaned aortae 1 week after tamoxifen administration showed that acute loss of *Prdm16* upregulated a similar profile of genes to that observed in cKO mice. This included synthetic marker genes such as *Ankrd1*, *Coro6*, *Chrdl1* and *Col14a1*, as well as *Tnnt2* and *Myo18b* (Fig. 2i and Extended Data Fig. 3f,g). GO analysis of transcripts upregulated in iKO versus control aortae identified processes related to synthetic SMC modulation, including migration, proliferation, adhesion and wound healing (Fig. 2j). Altogether, these findings demonstrate that PRDM16 is required to repress the synthetic gene program in SMCs and suggest a critical role for PRDM16 in maintaining the contractile phenotype of SMCs.

### Loss of PRDM16 promotes the development of SMC-rich, fibrous atherosclerotic plaques

**Extended Data Fig. 4:**
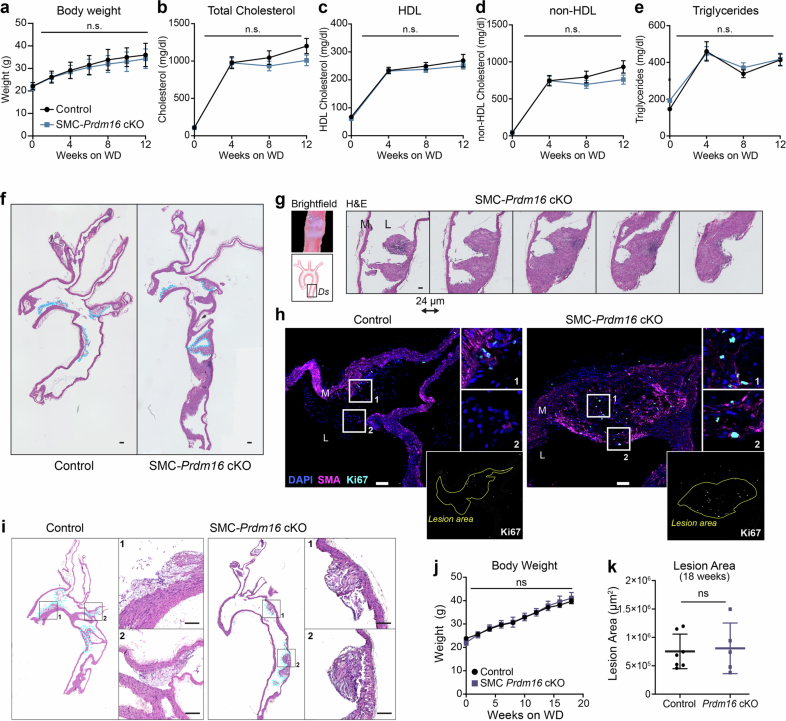
Loss of SMC-PRDM16 drives synthetic SMC modulation and fibrosis during atherogenesis. **a**. Body weights of control and cKO mice from the time of AAV8-Pcsk9-D377Y injection (8-weeks-old) and throughout 12 weeks of western diet feeding. Values represent mean ± S.E.M., n = 15/16 control/KO. **b–e**. FPLC measurements of total cholesterol (**b**) high-density lipoprotein (HDL) (**c**), non-high density lipoprotein fraction (non-HDL) (**d**) and triglyceride (**e**) in plasma samples from mice in (**a**). Values represent mean ± S.E.M, n = 15/16 control/KO. Multiple paired t-tests (1 per timepoint) were performed with Holm-Šídák correction for multiple testing. **f**. H&E-stained sections of aortae showing atherosclerotic lesions in control and cKO mice (lesion area outlined in blue). Scale bar: 100 µm. **g**. H&E staining of sequential sections through the same lesion from a cKO mice, Scale bar: 100 µm. **h**. Immunostaining for Ki67 (Cyan), ACTA2 (SMA, magenta), and DAPI (nuclei, blue) in lesions from control and cKO mice. Representative of n=3 per group. Scale bar: 50 µm. **i**. H&E staining of sagittal sections from the aortic arch of control and cKO mice at 18 weeks of Western diet feeding (lesion area outlined in blue). Scale bar: 100 µm. **j**. Body weights of control and cKO mice throughout 18 weeks of Western diet feeding. **k**. Total lesion area of control (n=7) and cKO (n=5) mice. M: tunica media, L: lumen. Source Data

To determine how PRDM16 loss in SMCs affects atherosclerotic lesion development, we induced atherosclerosis in control and SMC-cKO mice using AAV8-mediated delivery of mPCSK9 D377Y coupled with Western diet feeding under thermoneutral housing conditions (30 °C) for 12 weeks. Thermoneutral housing minimizes thermal stress, creating a more human-like physiological state by reducing metabolic rate. Control and SMC-cKO mice gained weight at similar rates (Extended Data Fig. 4a). Total plasma cholesterol and other plasma lipids (high-density lipoprotein, non-high-density lipoprotein and triglycerides) were comparably elevated in both groups (Extended Data Fig. 4b–e).

**Fig. 3:**
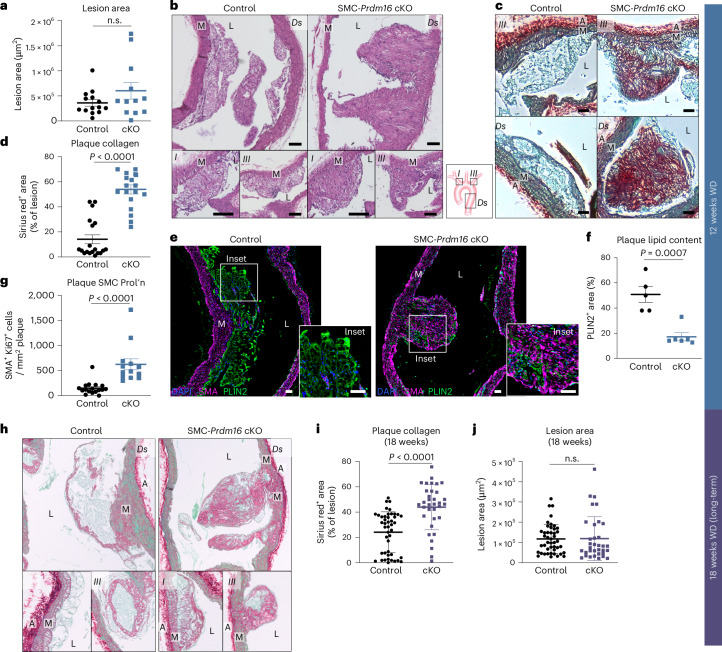
Loss of PRDM16 drives synthetic SMC modulation and fibrosis during atherogenesis. **a**, Lesion area in aortae from control and cKO mice injected with AAV8-*mPCSK9* D377Y followed by Western diet feeding for 12 weeks (averaged from 5 sequential sagittal sections spaced 24 µm apart). Values represent mean ± s.d., *n* = 14/12 control/KO. **b**, H&E-stained sagittal sections of aortic arch showing lesions in control and cKO mice. Representative of *n* = 14/12 control/cKO, Scale bar, 100 µm. Schematic of aortic arch: I, brachiocephalic artery; III, left subclavian artery; Ds, descending aorta. **c**, Sirius red/Fast-Green staining for collagen in aortic arch sections from control and cKO mice. Scale bar, 50 µm. **d**, Sirius-red-positive area per lesion area. Values represent mean ± s.e.m. Each dot represents a lesion, *n* = 19/18 control/cKO from *n* = 5/5 control/cKO mice. An unpaired, two-tailed *t*-test was performed, *P* < 0.0001 with an *F*-test for variance (n.s.). **e**, Immunostaining for PLIN2 (green), ACTA2 (SMA, magenta) and DAPI (nuclei, blue) in lesions from control and cKO mice. Scale bar, 50 µm. **f**, PLIN2 positive area per lesion area (*n* = 5/6 lesions of control and cKO mice. Values represent mean ± s.e.m. Each dot represents a lesion from three mice per group. An unpaired, two-tailed *t*-test was performed, *P* < 0.0001, with an *F*-test for variance (n.s.). **g**, Ki67 and ACTA2 double-positive cells per lesion area. Values represent mean ± s.e.m. Each dot represents a lesion, *n* = 17/13 for control/cKO mice. An unpaired, two-tailed *t*-test was performed, *P* < 0.0001, with an *F*-test for variance (n.s.). Prol’n, proliferation. **h**, Sirius red/Fast-Green staining for collagen in aortic arch of control and cKO mice injected with AAV8-*mPCSK9* D377Y and maintained on Western diet (WD) for 18 weeks. Scale bar, 50 µm. **i**, Sirius-red-positive area per lesion area. Data are represented as mean ± s.e.m. Each dot represents a lesion, *n* = 45/34 lesions from *n* = 7/5 mice for control/KO. An unpaired, two-tailed *t*-test was performed with an *F*-test for variance (n.s.). **j**, Individual lesion size. Values represent mean ± s.d. Each dot represents a lesion, *n* = 45/33 lesions from *n* = 5/7 control/cKO mice. An unpaired, two-tailed *t*-test with Welch’s correction was performed with an *F*-test for variance (*P* = 0.01). M, tunica media; L, lumen; n.s., not significant. Source data

We analyzed lesion development in serial sagittal sections of the aortic arch from control and SMC-cKO mice (Extended Data Fig. 4f). Atherosclerotic plaques developed in both groups, and total lesion area did not differ significantly between control and cKO mice, although variability was higher in the cKO group (Fig. 3a). Hematoxylin and eosin (H&E) staining revealed striking morphological differences between control and cKO lesions (Fig. 3b). Lesions in control mice displayed a typical fatty-streak morphology, with some showing a thin fibrous cap but generally not showing calcification or necrotic cores. By contrast, cKO lesions were extremely dense, disorganized and devoid of lipid-laden (foamy) cells (Fig. 3b and Extended Data Fig. 4g). Sirius red staining revealed that control lesions contained low amounts of collagen, which, when present, was primarily confined to the fibrous cap (Fig. 3c,d). By contrast, cKO lesions exhibited extensive collagen deposition, accounting for ~55% of lesion area compared with ~15% in controls (Fig. 3c,d). IF staining for smooth muscle actin (SMA) showed that cKO plaques contained much higher numbers and a wider distribution of SMCs compared with control plaques (Fig. 3e and Extended Data Fig. 4h). Conversely, cKO plaques had significantly fewer lipid-containing cells, marked by expression of Perilipin 2 (PLIN2) (Fig. 3e,f). We also assessed cell proliferation, a hallmark feature of SMC modulation. Control lesions contained few proliferating SMA^+^ cells, marked by Ki67 expression, which were confined to the media–lesion boundary (Fig. 3g and Extended Data Fig. 4h). By contrast, cKO lesions contained ~5-fold more proliferating SMA^+^ cells that were dispersed throughout the lesion (Fig. 3g and Extended Data Fig. 4h).

To examine later-stage disease, we extended Western diet feeding to 18 weeks. Body weight and total lesion area remained comparable between groups (Extended Data Fig. 4i–k). At this point, control plaques had evolved into more developed structures featuring more defined fibrous caps and increased collagen content (Fig. 3h,i). cKO lesions at 18 weeks exhibited a significant increase in Sirius-red-positive fibrotic area compared with controls but also contained more foam cells than cKO lesions at 12 weeks, indicating more advanced lesion progression (Fig. 3h,i). The size of individual lesions was similar between control and cKO mice at 18 weeks, suggesting that elevated proliferation in cKO SMCs did not drive continuous lesion enlargement (Fig. 3j). Altogether, these results demonstrate that *Prdm16* deficiency promotes the formation of fibroproliferative, SMC-rich lesions.

### Inducible loss of *Prdm16* in adult mice promotes the formation of fibrotic plaques with thicker caps

**Fig. 4:**
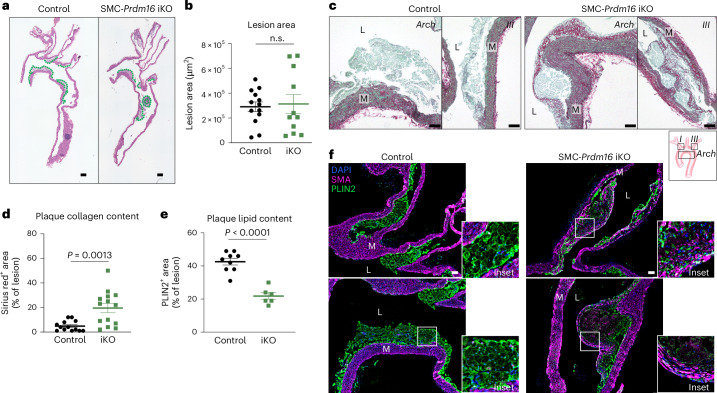
Acute loss of PRDM16 promotes synthetic SMC modulation and fibrosis. **a**, H&E staining of sagittal sections of aortic arch from control and SMC-iKO mice at 12 weeks of HFD feeding (lesion area outlined in green). Lesions in the arch, the start of the descending aorta and the beginnings of the branches were included. Scale bar, 100 µm. **b**, Lesion area (averaged from five sequential sagittal sections 24 µm apart per aorta). Values represent mean ± s.d., *n* = 13/11 from control/iKO mice. **c**, Sirius red/Fast-Green staining for collagen in aortic arches from control and iKO mice. Scale bar, 50 µm. **d**, Sirius-red-positive area per lesion area. Values represent mean ± s.e.m. Each dot represents a lesion, *n* = 13/15 lesions from *n* = 5/5 control/iKO mice. An unpaired, two-tailed *t*-test was performed with Welch’s correction, *P* = 0.0019 with an *F*-test for variance (*P* = 0.0002). **e**, PLIN2-positive area per lesion area in control and iKO mice. Values represent mean ± s.e.m. Each dot represents a lesion, *n* = 9/6 lesions from *n* = 3/3 control/iKO mice. An unpaired, two-tailed *t*-test was performed, *P* < 0.0001 with an *F*-test for variance (n.s.). **f**, Immunostaining for PLIN2 (green), ACTA2 (SMA, magenta) and DAPI (nuclei, blue) in lesions from control and iKO mice. Representative of *n* = 3 per group. Scale bar, 50 µm. M, tunica media, L, lumen. Source data

**Extended Data Fig. 5:**
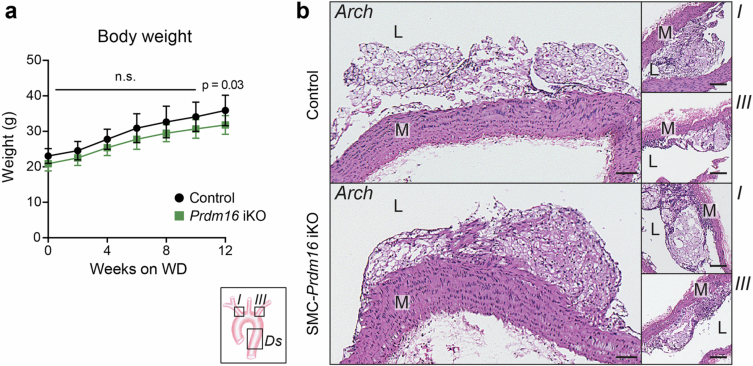
Inducible loss of *Prdm16* promotes the development of fibrous plaques. **a**. Body weights of control and iKO mice from the time of PCSK9 injection (8-weeks-old) and throughout 12 weeks of western diet feeding. Values represent mean ± S.E.M. (n = 15/13 control/KO). Multiple paired t-tests with Welch correction were performed with Holm-Šídák correction for multiple testing. **b**. H&E-stained sagittal sections of the aortic arch from control and iKO mice. Images are representative of n=13/11 control/KO. Scale bar: 50 µm. A diagram of the aortic arch shows the orientation: I= brachiocephalic artery, III = left subclavian artery, Ds = descending aorta. Source Data

To assess how *Prdm16* deletion in adult SMCs affects atherosclerosis, we induced plaque formation in control and SMC-iKO mice 1 week after tamoxifen treatment via injection of AAV8-*mPCSK9* D377Y and Western diet feeding for 12 weeks. Body weight trajectories were similar between the groups until 12 weeks, when iKO mice weighed slightly less than controls (Extended Data Fig. 5a). Total lesion area did not differ between control and iKO mice (Fig. 4a,b). H&E staining showed that iKO lesions contained fewer foam cells with smaller lipid droplets compared with control lesions (Extended Data Fig. 5b). Notably, iKO lesions developed prominent fibrous caps, while control lesions either lacked a cap or showed only a thin fibrous layer (Fig. 4c,f and Extended Data Fig. 5b). Sirius red staining showed that collagen content was significantly elevated in iKO plaques, with widespread collagen deposition throughout the plaque core and the fibrous cap region (Fig. 4c,d). IF analysis showed that iKO plaques contained higher SMC content accompanied by a reduction in PLIN2⁺ lipid-containing cells, supporting a shift toward a fibrotic and SMC-dominant plaque phenotype (Fig. 4e,f).

### Loss of *Prdm16* upregulates synthetic genes in SMCs and promotes synthetic SMC development during atherosclerosis

**Extended Data Fig. 6:**
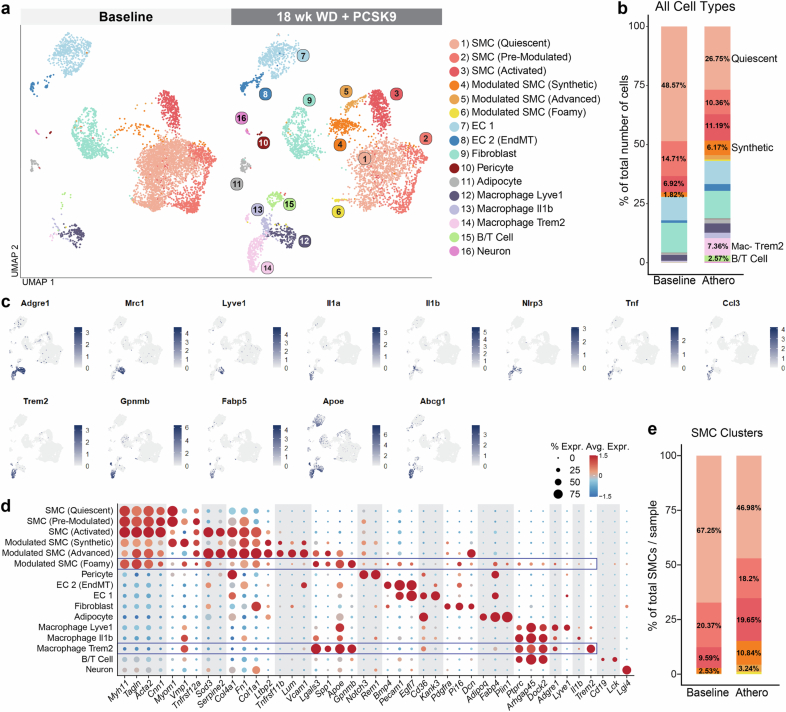
Atherosclerosis induces the development of multiple types of modulated SMCs. **a**. UMAP visualization of scRNAseq expression data from control aortae under homeostatic (11,297 cells, n=4 pooled) and atherogenic conditions (9,653 cells, n=4 pooled) (AAV8-PCSK9-D377Y injection and Western diet for 18 weeks). **b**. Bar charts representing the proportion of cells assigned to each cluster relative to the total number of cells. **c**. UMAP feature plots showing expression of immune and foam cell marker genes. **d**. Dot plot displaying the expression of cluster-defining genes. Blue boxes used to annotate the foamy modulated SMC- and the Macrophage Trem2 clusters. **e**. Bar charts representing the proportion of SMC types relative to the total number of SMCs. Source Data

To investigate the role of PRDM16 in SMC phenotypic modulation during advanced atherosclerosis, we used our native tissue scRNA-seq approach. We induced atherosclerosis in control and SMC-iKO mice harboring a tdTomato SMC lineage reporter gene via AAV8-mPCSK9 D377Y injection 1 week after tamoxifen treatment. After 18 weeks of feeding mice a Western diet, aortae were collected and processed for fixed-tissue scRNA-seq. We first performed unsupervised clustering of 14,856 vascular cells from control mice under baseline and atherogenic conditions. This analysis identified 6 cell clusters unique to atherosclerosis, in addition to the 11 baseline clusters (Extended Data Fig. 6a,b). As anticipated, atherosclerosis led to a marked expansion and diversification of immune cells (Extended Data Fig. 6a–c). Macrophages marked by expression of *Lyve1* and *Mrc1* (‘macrophage Lyve1’) were present under baseline and disease conditions. Atherogenic conditions induced the emergence of two additional macrophage clusters: ‘macrophage Il1b’ cells, expressing high levels of pro-inflammatory genes (*Il1b*, *Nlrp3*, *Tnf* and *Cxcl2*); and ‘macrophage Trem2’ cells, enriched for expression of lipid and cholesterol metabolism genes (*Trem2*, *Gpnmb*, *Lpl* and *Fabp5*), cholesterol exporters (*Apoe* and *Abcg1*) and foam cell markers (*Lgals3* and *Spp1*)^25–28^ (Extended Data Fig. 6c,d).

Atherosclerosis also profoundly altered the composition and phenotypic state of SMCs. Compared with baseline, diseased aortae showed an increase in activated SMCs and a reduction in quiescent SMCs (Extended Data Fig. 6b,e). Notably, three distinct subtypes of modulated SMCs emerged. These modulated clusters retained expression of quiescent SMC markers (*Myh11*, *Tagln* and *Acta2*), although at lower levels than in the baseline SMC populations (Extended Data Fig. 6d). ‘Synthetic modulated SMCs’ expressed high levels of synthetic and fibrotic cap marker genes, including *Col14a1*, *Fn1*, *Col1a1* and *Ltbp2* (Extended Data Fig. 6d). These cells also retained moderate expression of the quiescent and premodulated SMC marker *Myom1*. Many of the signature genes expressed in synthetic modulated SMCs overlapped with those in premodulated SMCs, suggesting that premodulated cells may transition into synthetic modulated SMCs during atherosclerosis. ‘Advanced modulated SMCs’ expressed elevated levels of fibrotic markers, as well as higher levels of *Lum*, *Tnfrsf11b*, *Vcam1* and the osteogenic marker *Spp1* (Extended Data Fig. 6d). Interestingly, advanced modulated SMCs and activated SMCs shared expression of certain markers such as *Sod3* and *Serpine2*, suggesting that activated SMCs may serve as precursors for advanced modulated SMCs. ‘Foamy modulated SMCs’ retained low expression of contractile genes but expressed lower levels of synthetic markers compared with advanced or synthetic modulated SMCs. These cells also expressed macrophage foam cell-associated genes such as *Apoe*, *Spp1* and *Lgals3*. However, these cells were distinct from the Trem2^+^ macrophage foam cell population, as they lacked expression of *Trem2* and immune markers such as *Ptprc* and *Arhgap45* (Extended Data Fig. 6c,d).

**Fig. 5:**
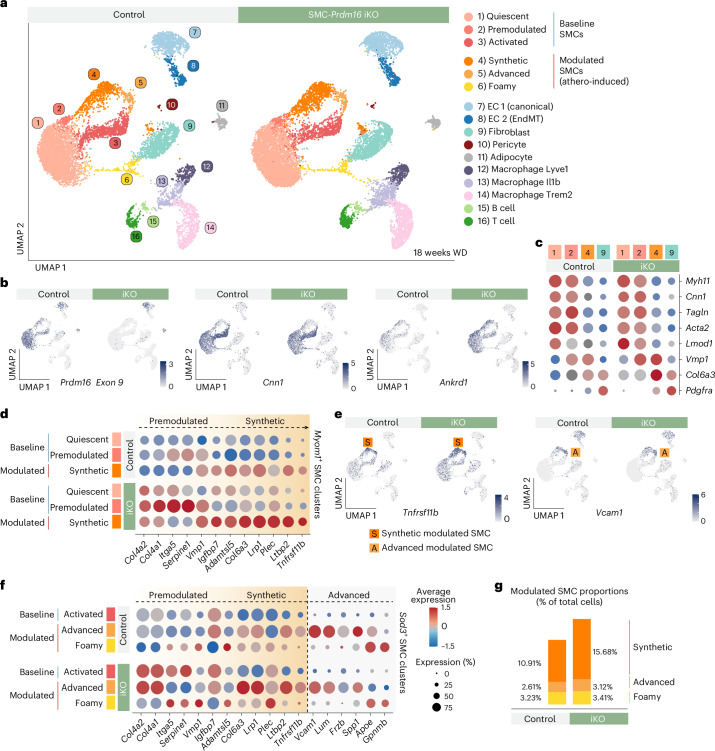
Loss of PRDM16 drives synthetic gene expression in all SMC populations and increases synthetic SMC content during atherosclerosis. **a**, UMAP visualization of scRNA-seq expression data from aortae of control (*n* = 4 (pooled), 9,653 cells) and SMC-iKO mice (*n* = 4 (pooled), 12,254 cells) injected with AAV8-*Pcsk9*-D377Y and fed a Western diet for 18 weeks. **b**, UMAP feature plots of *Cnn1, Prdm16* exon 9 and *Ankrd1* expression. **c**, Dot plot showing expression of: SMC contractile genes (cluster 1), *Vmp1* (marker of premodulated SMCs, cluster 2), *Col6a3* (marker of synthetic modulated SMCs, cluster 4) and *Pdgfra* (fibroblast marker, cluster 9). **d**, Dot plot showing expression of pre- and synthetic-modulation genes in the three *Myom1* + SMC clusters (quiescent, premodulated and synthetic modulated) from control and iKO aortae. **e**, UMAP feature plots showing expression of *Tnfrsf11b* (marker of pre- and synthetic modulation) and *Vcam1* (marker of advanced modulation), in control and iKO aortae. S, synthetic modulated SMCs; A, advanced modulated SMCs. **f**, Dot plot showing expression of (pre, synthetic and advanced) SMC modulation genes in the three *Sod3* + SMC clusters (activated, advanced and foamy) from control and iKO aortae. **g**, Bar chart showing the proportion of modulated SMC clusters as a percentage of the total SMC cells.

**Extended Data Fig. 7:**
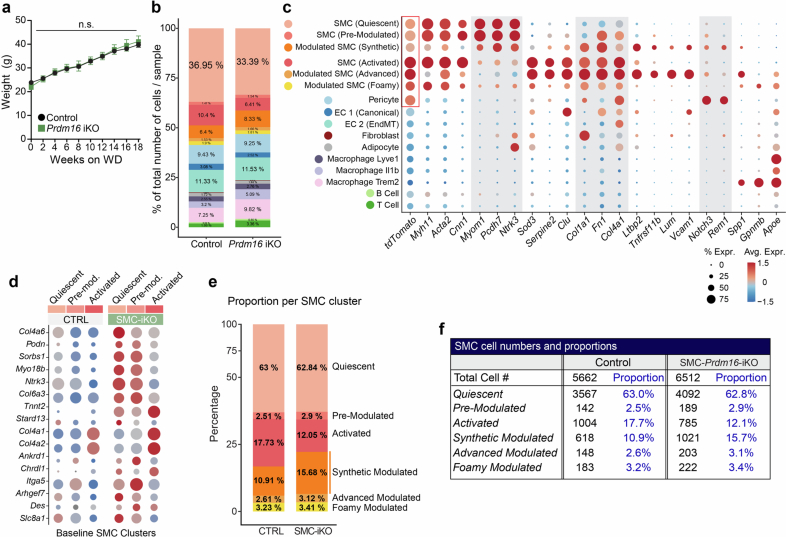
Loss of PRDM16 drives synthetic gene expression in all SMC populations and increases synthetic SMC content during atherosclerosis. **a**. Body weights of control and SMC-iKO mice injected with AAV8-PCSK9-D377Y and fed a Western diet for 18 weeks (n=4 per group). **b**. Bar charts representing the proportion of cells assigned to each cluster relative to the total number of cells. **c**. Dot plot showing the expression of SMC-cluster marker genes identified under atherogenic conditions. Red box indicates tdTomato+ (SMC-lineage+) cells. **d**. Dot plot showing expression of synthetic and circulatory development genes affected by PRDM16 in baseline SMC clusters from control and SMC-iKO aortae. **e**. Bar charts representing the proportion of the different SMC clusters relative to the total number of SMCs. **f**. Absolute number of cells in each (modulated) SMC cluster shown in e.

Next, we focused on the effects of *Prdm16* deficiency in SMCs during atherosclerosis. We performed unsupervised clustering of 21,907 cells from the aortae of control and iKO mice subjected to atherogenic conditions (Fig. 5a and Extended Data Fig. 7a,b). As noted earlier, all mice included a tdTomato lineage reporter to enable identification of SMC-derived cells. Using custom probes, *tdTomato* expression was detected across all SMC populations, including the three modulated clusters, confirming their derivation from *Myh11* + SMCs (Extended Data Fig. 7c). In control aortae, *Prdm16* was expressed in quiescent, premodulated and activated SMC clusters and was downregulated in all modulated SMC populations. In iKO aortae, *Prdm16* expression was specifically ablated in all SMCs while remaining intact in ECs and adipocytes (Fig. 5a,b). *Prdm16*-iKO did not affect the expression of SMC contractile genes such as *Cnn1*, *Myh11* and *Tagln*, and these genes were downregulated to a similar extent in iKO and control mice during SMC modulation (Fig. 5b,c). Loss of *Prdm16* upregulated synthetic genes, such as *Ankrd1*, *Des* and *Sorbs1*, in all baseline SMC clusters (quiescent, premodulated and activated) (Fig. 5a,b and Extended Data Fig. 7d). In addition, genes associated with premodulation and synthetic modulation, including *Ankrd1*, *Col4a2*, *Plec* and *Tnfrsf11b*, were expressed at higher levels in all SMC clusters from iKO aortae (Fig. 5b–e). The loss of *Prdm16* did not affect the expression of marker genes of advanced or foamy SMCs (*Vcam1*, *Spp1* and *Apoe*) (Fig. 5e,f). Notably, *Prdm16* deletion not only upregulated synthetic gene expression in SMCs but also increased the proportion of synthetic modulated SMCs by ~1.5-fold (Fig. 5g and Extended Data Fig. 7e,f). In summary, *Prdm16* deficiency shifts SMCs toward a more synthetic/fibrotic phenotype and enhances the formation of synthetic modulated SMCs. These data further show that PRDM16 selectively restrains synthetic SMC identity, without influencing SMC progression toward advanced or foamy modulated states.

### PRDM16 represses SMC synthetic processes and fibrosis

**Fig. 6:**
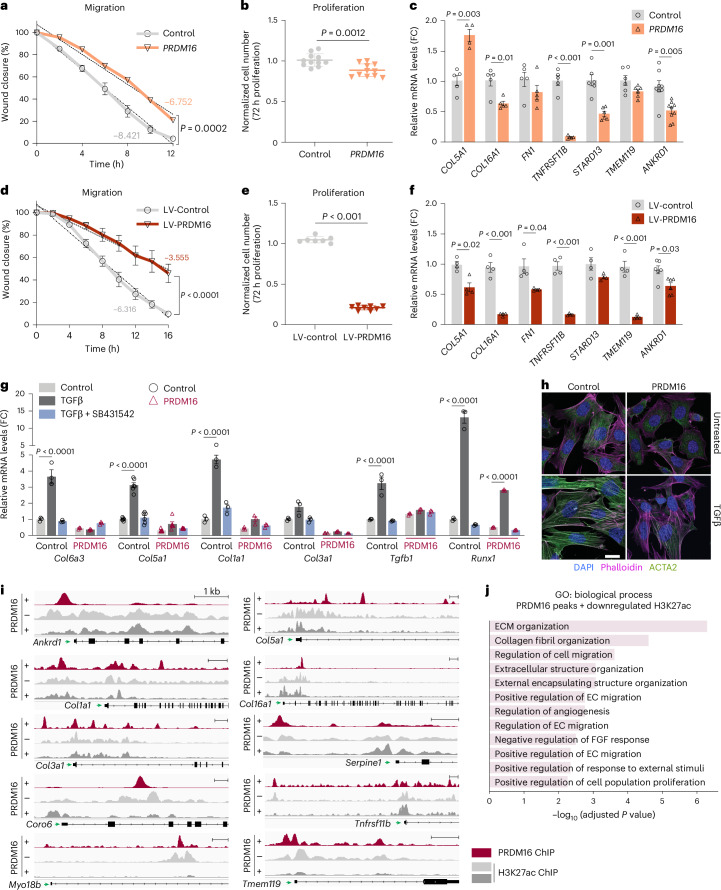
PRDM16 expression represses SMC synthetic processes and fibrosis. **a**, Migration of hCaSMCs expressing either a control CRISPRa or a CRISPRa guide to activate *PRDM16*. Scratch closure was quantified by microscopy every 2 h. Values represent mean ± s.e.m., *n* = 6 biological replicates per group. Regression slopes were significantly different between groups (*F*(1,59) = 15.39, *P* = 0.0002). **b**, MTT assay for cell proliferation in cells from **a**. Values represent mean ± s.e.m., *n* = 11 biological replicates per group. An unpaired, two-tailed *t*-test was performed, *P* = 0.0012 with an *F*-test for variance (n.s.). **c**, mRNA levels of indicated genes in control and *PRDM16*-CRISPRa cells. Values represent mean ± s.e.m., *n* > 5 biological replicates per group. Multiple paired *t*-tests were performed with Holm–Šídák correction for multiple testing. **d**, Quantification of migration of hCaSMCs transduced with control or PRDM16-expressing lentivirus. Scratch closure was quantified every 2 h. Values represent mean ± s.e.m., *n* = 5 biological replicates per group. Regression slopes were significantly different between groups (*F*(1,86) = 38.88, *P* < 0.0001. **e**, MTT assay for cell proliferation in control and lentiviral (LV) PRDM16-expressing cells. Values represent mean ± s.e.m., *n* = 7/8 biological replicates for control/PRDM16. An unpaired, two-tailed *t*-test was performed, *P* < 0.0001, with an *F*-test for variance (n.s.). **f**, mRNA levels of indicated genes in control and LV-PRDM16-expressing hCaSMCs. Values represent mean ± s.e.m., *n* > 4 biological replicates. Multiple paired *t*-tests were performed with Holm–Šídák correction for multiple testing. **g**, mRNA levels of indicated genes in control and PRDM16-expressing fibroblasts treated with TGFβ1 and/or SB431542 or vehicle control for 48 h. Values represent mean ± s.e.m., *n* ≥ 3 biological replicates per condition. One-way ANOVA with Holm–Šídák correction. Normality confirmed by Kolmogorov–Smirnov test. **h**, Immunostaining for phalloidin (magenta), ACTA2 (SMA, green) and DAPI (nuclei, blue) in control and PRDM16-expressing cells treated with or without TGFβ1 for 48 h (representative of *n* = 3 biological replicates). Scale bar, 10 µm. **i**, ChIP-seq tracks for PRDM16 and H3K27-Ac at indicated synthetic genes in control and PRDM16-expressing fibroblasts. **j**, GO analysis of genes with PRDM16 ChIP-seq peaks that also display a concomitant decrease of H3K27-Ac in PRDM16-expressing versus control cells. Source data

**Extended Data Fig. 8:**
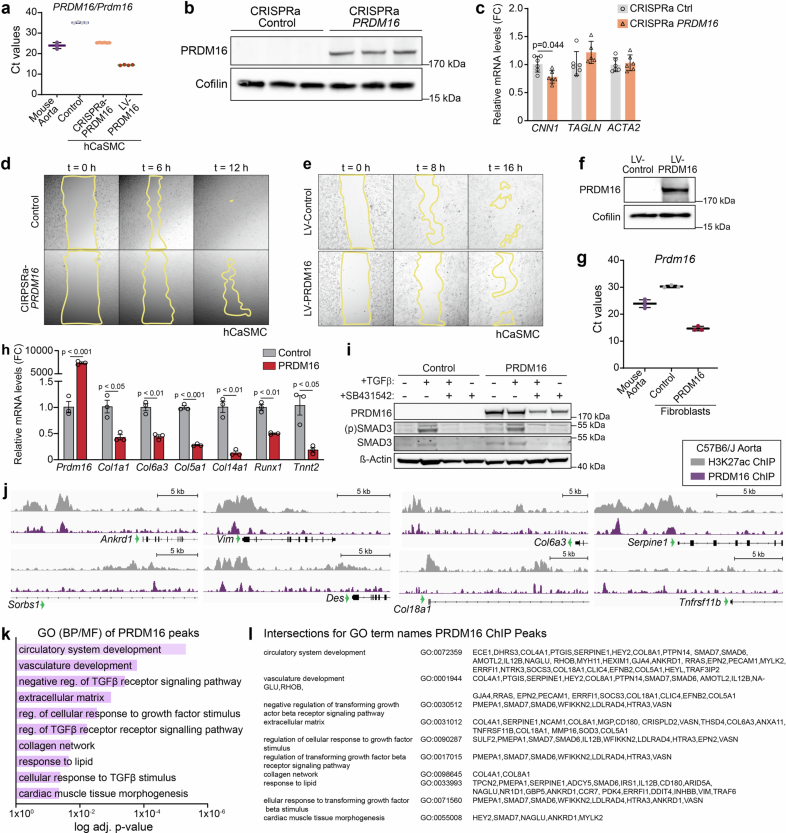
PRDM16 expression represses SMC synthetic processes and fibrosis. **a**. Ct values for PRDM16/Prdm16 mRNA levels in mouse aorta, hCaSMCs, and hCaSMCs transduced with PRDM16-CRISPRa or PRDM16 lentivirus (LV). **b**. Immunoblot showing PRDM16 protein levels in control and PRDM16-CRISPRa hCaSMCs. Cofilin was used as a loading control (n=3 per condition). **c**. mRNA levels of indicated genes in control and PRDM16-CRISPRa hCaSMCs. **d**. Images of the scratch-migration assay from Fig. 6a. The yellow lines mark the wound borders. **e**. Images from the scratch assay in Fig. 6d. The yellow lines mark the wound borders. **f**. immunoblot showing PRDM16 protein levels in control and LV-PRDM16 expressing hCaSMCs. Representative of n=3. Cofilin was used as a loading control. **g**. Ct values for Prdm16 mRNA in mouse aorta, mouse fibroblasts and fibroblasts expressing PRDM16. **h**. mRNA levels of indicated genes in control and PRDM16-expressing fibroblasts. Values represent mean ± S.E.M. (n = 3 biol. repl.). **i**. Immunoblot for PRDM16, SMAD3 and phospho-SMAD3 (pSMAD3) in PRDM16-expressing and control cells ± TGFb and/or SB431542. β-actin was used as a loading control. Image is representative of n = 3. **j**. ChIP tracks for PRDM16 and H3K27-Ac in aortae from C57B6/J mice. **k**. GO analysis of genes with a PRDM16 ChIP-seq peak. **l**. Intersection of GO analysis with ChIP-seq tracks: each gene entry corresponds to an identified ChIP-seq peak and associated GO terms.

To evaluate the role of PRDM16 in human SMCs, we utilized human coronary artery SMCs (hCaSMCs). Notably, *PRDM16* expression was lost in cultured SMCs, providing a platform for gain-of-function assays (Extended Data Fig. 8a). We induced *PRDM16* expression in hCaSMCs using CRISPR activation (CRISPRa) with a guide targeting the *PRDM16* promoter. This approach increased *PRDM16* expression from near undetectable levels (Ct >35) to physiologic levels (Ct ~25), comparable to its endogenous expression levels in mouse aortae (Extended Data Fig. 8a,b). *PRDM16* activation did not affect the expression of SMC marker genes *TAGLN* and *ACTA2* and only slightly decreased expression of *CNN1* (Extended Data Fig. 8c). A key feature of synthetic SMC modulation during atherosclerosis is the migration of SMCs out of the media to form a fibrous cap. We tested the role of PRDM16 in SMC migration using a scratch assay. Cells were grown to confluence, followed by inducing a wound and imaging migration with time-lapse microscopy. *PRDM16* activation significantly reduced the wound closure rate by about 20% (Fig. 6a and Extended Data Fig. 8d). *PRDM16*-CRISPRa cells also displayed a small but significant decrease in proliferation (Fig. 6b) and expressed lower levels of multiple synthetic/fibrotic genes (that were also upregulated in *Prdm16-*KO aortae), including *COL16A1*, *TNFRSF11B*, *STARD13* and *ANKRD1* (Fig. 6c). To assess the effects of stronger PRDM16 induction, we transduced hCaSMCs with a lentiviral vector encoding PRDM16. The higher expression of *PRDM16* resulted in even greater suppression of synthetic behaviors: migration decreased by ~50%, proliferation decreased by ~70% and synthetic/fibrotic gene expression was broadly repressed (Fig. 6d–f and Extended Data Fig. 8a,e,f). These results indicate that PRDM16 suppresses human SMC synthetic processes in a dose-dependent manner.

We extended these findings to fibroblasts, which naturally exhibit a highly synthetic phenotype and lack *Prdm16* expression (Extended Data Fig. 8g). Lentiviral expression of PRDM16 downregulated synthetic genes, and other genes that were also repressed by PRDM16 in aortae (from RNA-seq) (Fig. 6g and Extended Data Fig. 8h). To further assess fibrosis, we treated cells with the canonical pro-fibrotic cytokine TGFβ1. TGFβ1 markedly upregulated synthetic genes, including multiple collagen genes and the epithelial to mesenchymal transition regulator *Runx1* by 3- to 15-fold; these effects were fully attenuated by the TGFβ receptor inhibitor SB431542. Remarkably, PRDM16 completely suppressed synthetic genes at baseline and nearly completely abolished the fibrotic gene response to TGFβ1 (Fig. 6g). TGFβ1 induced a dramatic remodeling of the actin cytoskeleton in control cells, leading to the formation of prominent stress fibers. This effect was nearly completely blocked in PRDM16-expressing cells (Fig. 6h). Interestingly, PRDM16 did not interfere with TGFβ1-induced SMAD3 phosphorylation, suggesting that PRDM16 inhibits TGFβ signaling at the transcriptional level rather than by blocking receptor activation (Extended Data Fig. 8i). Collectively, these data indicate that PRDM16 potently suppresses fibrosis, even in highly synthetic cell types.

To understand how PRDM16 represses the synthetic gene program, we analyzed chromatin immunoprecipitation sequencing (ChIP-seq) data from PRDM16-expressing fibroblasts^29^. PRDM16 binding peaks were identified at many synthetic genes that were transcriptionally regulated by PRDM16 in mouse SMCs and human SMCs, including *ANKRD1*, *SORBS1* and *DES* (Fig. 6i). These binding sites displayed reduced H3K27-acetylation levels upon PRDM16 expression, indicating functional repression (Fig. 6i). Genes located near PRDM16-binding sites that displayed PRDM16-mediated decreases in H3K27-acetylation were enriched in pathways related to fibrosis, ECM organization and cell migration (Fig. 6j). ChIP for endogenous PRDM16 in mouse aortae similarly identified binding sites at many synthetic genes such as *Ankrd1*, *Serpine1*, *Des*, *Tnfrsf11b*, *Sorbs1*, *Vim*, *Col4a1*, *Col8a1* and *Col5a1* (Extended Data Fig. 8j). GO analysis of the PRDM16 binding regions in aortae converged on fibrotic processes, similar to those upregulated in *Prdm16* KO aortae (Extended Data Fig. 8k,l). Taken together, these results demonstrate that PRDM16 binds to chromatin and represses transcription of synthetic genes.

## Discussion

This study identifies the CVD-associated gene *PRDM16* as a critical regulator of SMC identity. Our findings demonstrate that PRDM16 suppresses the synthetic SMC gene program and restrains the phenotypic switch toward synthetic and fibrotic SMC states, without influencing other SMC fate transitions. *PRDM16* is downregulated during SMC modulation in atherosclerosis, and genetic loss of *Prdm16* in mice results in the development of fibrous lesions with reduced lipid content.

The pathways that downregulate *PRDM16* expression during atherosclerosis are unknown but of substantial interest. While PRDM16 has been extensively studied in other contexts, few regulators of *PRDM16* expression have been reported. Atherosclerotic lesions are dynamic microenvironments that are influenced by multiple pathways. Altered hemodynamics, immune cell infiltration, ECM remodeling and vascular stiffness could all contribute to *PRDM16* downregulation and subsequent synthetic SMC phenotypic modulation.

Single-cell analyses revealed that PRDM16 represses synthetic gene expression across all SMC populations, both at baseline and under atherogenic conditions. In baseline SMC clusters, loss of PRDM16 upregulated synthetic genes without affecting contractile gene expression. In addition, deletion of *Prdm16* in adult SMCs increased the proportion of cells undergoing atherosclerosis-associated synthetic modulation. The enhanced fibrotic profile and increased fibrous cap formation observed in lesions from *Prdm16*-deficient mice are phenotypic features associated with a lower risk of rupture in human atherosclerosis. Interestingly, we observed a stronger fibrotic phenotype in plaques from cKO (*Tagln-Cre*) compared with iKO (*Myh11-Cre-ERT2*) mice, despite a similar alteration of the gene program in both models. The more pronounced fibrosis in cKO mice may stem from early developmental loss of PRDM16 and accumulated changes in the ECM and/or may be influenced by *Prdm16* deletion in other cell types, such as cardiac muscle, that are also targeted by *Tagln-Cre* during development^31^.

In addition to identifying synthetic modulated and advanced modulated SMCs with features consistent with those reported in prior scRNA-seq studies^32,33^, our fixed-tissue single-cell approach uncovered previously unrecognized SMC clusters, including premodulated SMCs, which may represent precursors to synthetic modulated SMCs. We also identified three distinct modulated SMC states in atherosclerotic aortae: fibrotic, advanced and foamy. There has been debate in the literature regarding the presence of foamy/macrophage-like SMCs based on results from recent scRNA-seq analyses^32–36^. We speculate that the immediate fixation of aortae after dissection enabled the capture of cell types that are difficult to detect using other methods. This may be attributed to several factors: (1) fixation preserves the transcriptional state of cells within their native tissue environment, minimizing artifacts introduced by tissue processing and cell isolation; (2) fixation may stabilize fragile cell populations that are otherwise lost during dissociation; and (3) the probe-based sequencing approach affords increased depth, enhancing resolution and detection sensitivity.

PRDM16 powerfully regulates cellular metabolism in several cell types. In adipose and intestinal cells, PRDM16 interacts with members of the PPAR transcription factor family to directly activate the expression of fatty acid oxidation genes and drive fatty acid oxidation^17–19^. PRDM16 also promotes mitochondrial biogenesis and dynamics in adipocytes, cardiomyocytes and hematopoietic stem cells via a direct transcriptional pathway^20,37^. Interestingly, altered PRDM16 levels in SMCs did not affect the expression of metabolic or mitochondrial genes, suggesting that PRDM16 represses synthetic SMC activity via pathways distinct from its known role in metabolic regulation. ChIP experiments show that PRDM16 binds to chromatin at many synthetic/fibrotic genes, suggesting direct repression of this gene program in SMCs. PRDM16 can repress gene transcription via interacting with various complexes, including CtBP^19^, the CBX4–polycomb complex^37^ and EHMT1/2^38–40^. Further studies will be needed to clarify the essential PRDM16-complex components and interacting partners in SMCs.

Previous studies have identified several transcriptional regulators of SMC modulation, most notably TCF21, KLF4 and OCT4. *Tcf21* is upregulated in synthetic modulated SMCs, where it promotes the expression of fibroblast and synthetic genes. *Tcf21* deletion in mouse models reduces both the number of modulated SMCs and overall lesion burden^32^. KLF4 and OCT4, both pluripotency-associated factors, are upregulated during SMC modulation but have contrasting roles. KLF4 suppresses contractile SMC marker genes and promotes the expression of pro-inflammatory mediators, while OCT4 supports contractile identity and enhances lesion stability^41,42^. The loss of *Klf4* or *Tcf21* leads to smaller atherosclerotic lesions, whereas deletion of *Oct4* increases lesion size. These phenotypes differ from those observed in *Prdm16*-deficient models, in which SMCs are preferentially driven toward a synthetic phenotype without altering lesion size. Interestingly, PRDM16 restrained SMC synthetic modulation without affecting the expression levels of *Tcf21* or *Klf4*, although whether PRDM16 modulates their transcriptional activity remains to be determined.

SMC phenotypic switching is implicated in other vascular diseases beyond atherosclerosis, such as aortic aneurysms and dissections. Notably, a recent study found that *Prdm16* ablation aggravates elastase-induced aneurysms in mice, through upregulation of ADAM12 and inflammatory signaling^43^. By contrast, our SMC-specific *Prdm16* KO models did not show increased expression of *Adam12* or inflammatory markers such as *Cd68* and *Lgals3*. However, PRDM16 did downregulate expression of tumor necrosis factor receptor superfamily gene *Tnfrsf11b*. In atherosclerosis, TNFRSF11b is involved in ECM remodeling and repression of vascular calcification, rather than driving inflammation^32,44^. These findings suggest that PRDM16-mediated modulation of SMC fibrosis may interact with inflammation and matrix degradation pathways under aneurysm-inducing conditions.

We chose an AAV8-based approach to overexpress the PCSK9 gain-of-function mutant D377Y to induce hypercholesterolemia, allowing us to evaluate the impact of PRDM16 ablation at baseline and during lesion development within the same mouse strains. Since their introduction over a decade ago, AAV8-*PCSK9*-based models have been used to study atherosclerosis^45–47^. While commonly used models, including AAV8-PCSK9, *Ldlr⁻/⁻*, *ApoE⁻/⁻* and APOE3-Leiden CETP, share the limitation of not developing spontaneous plaque rupture^45,48^, they differ in disease kinetics and plaque composition and can thus be used to study different aspects of the disease. Some studies have reported reduced necrotic core formation in PCSK9-induced lesions compared to *ApoE⁻/⁻* mice^49^, although these findings are not universal and could be due to differences in virus titer or quality^45,47^. To further study the role PRDM16-regulated SMC modulation in models that may more closely reflect features of plaque vulnerability—such as intraplaque angiogenesis, hemorrhage or large necrotic cores—future studies could use a vein graft model^50^, introduce the *Fbn*^*1C1039G*^ mutation^51^ or extend high-fat diet feeding on the *ApoE⁻/⁻* background^45,49^.

PRDM16 is also expressed in ECs, albeit at lower levels than in SMCs. Interestingly, PRDM16 activity in ECs—but not SMCs—is required for arterial flow recovery following limb ischemia^26^, indicating that PRDM16 is dispensable for arteriogenesis in SMCs. Given the overlap between synthetic SMC modulation and endothelial-to-mesenchymal transition, it is plausible that PRDM16 may act to suppress endothelial-to-mesenchymal transition.

In conclusion, PRDM16 functions as a transcriptional gatekeeper of the synthetic program in SMCs. Inhibiting PRDM16 to enhance synthetic SMC activity and fibrous cap development could represent a promising strategy to promote plaque stability and reduce the risk of life-threatening atherothrombotic events.

## Methods

### Mice

Experiments were performed according to procedures approved by the University of Pennsylvania Institutional Animal Care and Use Committee (protocol 805649). All mice for this study were male and on a C57BL6/J background. *Prdm16*^*loxP/loxP*^ (B6.129-Prdm16tm1.1Brsp/J, RRID: IMSR_JAX:024992) mice were crossed with either *Tagln-Cre* (B6.Cg-Tg(Tagln-cre)1Her/J, RRID:IMSR_JAX:017491) or *Myh11-CreER*^*T2*^ (B6.FVB-Tg(Myh11-icre/ERT2)1Soff/J, strain number 019079, RRID:IMSR_JAX:019079) mice. Littermate floxed mice lacking Cre expression were used as controls. Mice were maintained on a 12-h light/dark cycle at room temperature (22 °C) and fed standard chow (LabDiet 5010) unless otherwise noted. For atherosclerosis experiments, mice were transferred to thermoneutral conditions (30 °C) at weaning. For iKO experiments, tamoxifen (Sigma, T5648) was injected intraperitoneally at a dose of 100 mg kg^−1^ for 4 days. To induce atherosclerosis, mice were retro-orbitally injected with AAV8-PCSK9 D377Y (Vector Biolabs) at a dose of 5 × 10^11^ genome copies per mouse, as described by Bjørklund et al.^47^. Following injection, mice were fed a Western diet (Research Diets D12079B). Body weights were measured biweekly, and plasma samples were collected every 4 weeks after a 3-h fasting period for lipid measurements.

For oral glucose tolerance tests, mice were fasted for 16 h before administering glucose (2 g kg^−1^) by oral gavage. Blood glucose levels were measured from tail vein blood at 0, 15, 30, 45, 60 and 90 min using an Ascensia Contour Next glucometer (Medline). For blood pressure measurements, a Visitech BP-2000 series II arterial pressure analysis system was used. Starting at 8 weeks of age, mice were trained daily for 3–5 days until consistent measurements were obtained. Each day, three to ten individual measurements were recorded and averaged over a 5-day period. Measurements were excluded if at least three consistent reads were not obtained.

### Histology and staining

Mice were euthanized, and the aorta was perfused through the left ventricle with phosphate-buffered saline (PBS). The aortic arch was isolated and fixed in 1% paraformaldehyde for 16 h, followed by four washes in PBS. Samples were then dehydrated and paraffin embedded. Longitudinal sections (6 µm) spaced 24 µm apart were stained with H&E. Before plaque area analysis, images were randomized and blinded. Elastic fibers were visualized using Verhoeff van Gieson staining (Polysciences), and collagen content was assessed by Sirius red staining counterstained with Fast Green. Collagen-positive area within plaques was quantified by selecting the ‘color range’ option in Adobe Photoshop and calculated as pixels per lesion area. For IF, slides were deparaffinized and antigen retrieval was performed in Bull’s Eye Decloaking buffer (Biocare). Primary antibodies (Supplementary Table 1) were incubated on sections overnight, followed by detection with secondary antibodies and t tyramide signal amplification (Akoya Biosciences). Slides were imaged on a Zeiss Axio Observer 7 or a Leica SP8 Confocal Microscope.

### Cell culture, migration assays and analyses

hCaSMCs (Cell Applications; #350-05a) were immortalized by transduction with hTERT-IRES-hygro (Addgene #85140) lentivirus^50^ and maintained in SMC basal medium supplemented with a complementPack (C-22162, PromoCell). The cells retained a SMC gene signature, comparable to primary SMCs^51^. Transcriptional activation of *PRDM16* was achieved using the synergistic activation mediator (SAM) complex. Guide RNAs were designed using CRISPick^52^, CHOPCHOP^53^ and the guidelines outlined by Nageshwaran et al.^54^. The *PRDM16* guide RNA (5′-GCAATCTGACACCCCTCGCCG-3′) was cloned into lentiSAMv2 (Addgene #75112). hCASMC-hTert cells were transduced with lentiSAMv2 and lentiMPHv2 (Addgene #89308) in 8 µg ml^−1^ polybrene, followed by selection with Blasticidin (Invivogen; 10 μg ml^−1^) and Hygromycin (Gibco; 400 μg ml^−1^). For lentiviral PRDM16 expression, a HA-tagged *Prdm16* cDNA (CCDS71532) was cloned into pLentiV2-CMV Puro (Addgene #17448). hCaSMCs were transduced with lentivirus in the presence of 8 µg ml^−1^ polybrene and selected with 2.5 µg ml^−1^ puromycin (Sigma). Where indicated, cells were treated with mouse TGFβ1 (R&D Systems) at 5 ng ml^−1^, and SB431542 (Invivogen) at 10 µM. For migration assays, cells were seeded to confluence on µ-slides (Ibidi) coated with 5 µg ml^−1^ fibronectin (Corning). The next day, the cells were pretreated for 2 h with 2 µM Mitomycin C (Sigma). A scratch was created and scratch closure was monitored every 10 min for 14 h at 37 °C and 5% CO_2_ using an EVOS microscope (Thermo Fisher). Scratch area was quantified using ImageJ software. For proliferation measurements, cells were seeded at 10% confluency and grown for 72 h, and cell density was assessed using a 3-(4,5-dimethylthiazol-2-yl)-2,5-diphenyltetrazolium bromide (MTT) assay (ThermoFisher).

### RNA isolation, RT–qPCR and bulk RNA-seq analyses

Total RNA was extracted using TRIzol (Invitrogen), followed by RNA purification using PureLink RNA columns (ThermoFisher). cDNA was synthesized using the MultiScribe Reverse Transcriptase kit (Thermo Scientific). Real-time quantitative PCR (RT–qPCR) was performed on an ABI7900HT PCR machine with SYBR green (Applied Biosystems) (primers in Supplementary Table 2). Relative gene expression changes were calculated using the ΔΔCT method, with TATA-box binding protein (*Tbp*) serving as the housekeeping gene.

For bulk RNA-seq analyses, aortae were isolated, PVAT and adventitia were removed, aortae were cut open and the endothelial layer was gently brushed off. RNA was extracted using the PicoPure RNA isolation kit (ThermoFisher). Next-generation sequencing was performed by Genewiz using the NovaSeq 6000 analyzer. Fastq files were aligned to GRCm39 using kallisto (version 0.44.0) with default parameters. The abundance.tsv files were read into R (version 4.3.0) using the tximport package (version 1.28.0) with the parameter ‘txOut = F’, to obtain gene-level quantification. The ComBat_seq function from the package sva (version 3.35.2) was utilized to correct the raw counts expression matrix from batch effects. The corrected expression matrix was converted to a differential gene expression object with the edgeR package (version 3.42.4). Lowly expressed genes were filtered out. Normalization factors were calculated to account for differences in sequencing depth across samples using the trimmed mean of M-values (TMM) method. Data were transformed to log_2_ counts per million for visualization. For differential gene expression analysis, the raw un-batch-corrected data were analyzed with the limma package (version 3.56.2). The data were converted using voomWithQualityWeights to apply voom transformation. A linear model with robust M-estimation was fit to minimize the effect of outliers. Empirical Bayes moderation was applied to the data to calculate statistics. Minus-average (MA) plots were constructed using ggplot2 (3.5.1). Gene set enrichment analysis was performed using the fgsea package (version 1.26.0) on the differentially expressed gene list, which was ordered by ‘-log_10_(*P* value) × sign(logFC)’ (FC, fold change) with the reference gmt files obtained from MSigDB (https://www.gsea-msigdb.org/gsea/msigdb/human/collections.jsp). Functional enrichment analysis was performed using the gprofiler2 package (version 0.2.3). Heatmaps were created using the ComplexHeatmap package (version 2.16.0), with the data scaled by converting to *Z* scores.

### scRNA-seq sample preparation and analyses

scRNA-seq was performed using the 10x Flex Platform with the Chromium Next GEM Single Cell Fixed RNA Sample Preparation Kit (1000414). Dissected aortae (root to the iliac bifurcation) were washed twice in cold RPMI, sliced into 1-mm rings on a chilled glass slide and fixed in 1 ml fixation solution (4% paraformaldehyde with 10× concentrated fixation/permeabilization buffer) per 25 mg tissue for 16 h at 4 °C. Tissue pieces were then centrifuged at 850*g* for 5 min, washed with 2 ml cold PBS, resuspended in 1 ml quenching buffer with 0.1 volume of Enhancer and adjusted to a final glycerol concentration of 10% before being stored at −80 °C. For digestion, aortae were processed individually in 2 ml of prewarmed RPMI 1640 containing 1 mg ml^−1^ Liberase TM (Roche, 05401020001) using a Miltenyi GentleMACS at 37 °C with the following program: spin at 50 rpm for 20 min, followed by two cycles of spinning at 1,000 rpm for 30 s and −1,000 rpm for 30 s. Tissue suspensions were pooled and filtered through a 30-µm strainer. Cells were centrifuged at 1,200*g* for 10 min and resuspended in 250 µl quenching buffer. A 5% aliquot was taken for DAPI staining and cell counting. Probe hybridization and cell capture were performed using the 10x Chromium Fixed RNA Kit; Mouse Transcriptome kit (1000495) according to the manufacturer’s instructions except for an increase in centrifugation speed (1,200*g* for 10 min). Custom probes were designed following 10x Genomics guidelines and ordered as oPools from IDT DNA Technologies.

For analyses, Fastq files were aligned to the mouse genome (mm10) with cellranger (version 7.2.0) using the command cellranger multi, which was aligned to a customized chromium mouse transcriptome probe set (version 1.0.1) containing manually designed probes for *Prdm16* Exon 9 and/or *TdTomato* (lineage tracer). The raw matrix files were read into R (version 4.3.0) and processed with DropletUtils (version 1.20.0) where droplets with a false discovery rate less than or equal to 0.01 were retained. These filtered datasets were then processed with SoupX (version 1.6.2) using autoEstCont and adjustCount features to estimate the contamination fraction and then adjust the expression matrices. We then used Seurat (version 4.3.0) for quality control processing, filtering and integration. The function CreateSeuratObject with the parameters ‘min.features = 20, min.cells = 100’ was used to make Seurat objects. Light filtering of the datasets was performed with the following parameters: ‘nCount_RNA > 500 & nCount_RNA < 20000 & nFeature_RNA > 150 & nFeature_RNA < 8000 & percent.mt < 10’. Sample counts were scaled for each cell by the total number of molecules detected and then multiplied by 10,000 and log transformed. The top 2,000 most variable features were identified in each dataset using the ‘vst’ selection method. Gene expression data were then scaled and centered across all cells while regressing out the mitochondrial percentage. Principal component analysis (PCA) was performed where dimensions 1 through 15 were utilized. A *k*-nearest neighbor graph was constructed, and clustering was performed on the nearest-neighbor graph using a Louvain algorithm and a resolution of 0.2. The processed Seurat objects were run through scDblFinder (version 2.0.3) where cells were identified as singlets or doublets. We recreated the starting Seurat objects with all cells that were identified as singlets, and refiltered the data with stricter quality control parameters of ‘nCount_RNA > 500 & nCount_RNA < 10000 & nFeature_RNA > 500 & percent.mt <= 1’. The top 2,000 variable genes shared across datasets were selected, and common reference points were identified between datasets using a canonical correlation analysis and log normalization method. The data for each experiment (knockout (KO) versus control) was integrated together, and the subsequent dataset’s expression was scaled and centered for each gene across all cells while regressing on the mitochondrial percentage, S.Score and G2M score. Harmony (version 1.2.0) was run on the integrated data for batch-effect correction; however, its effects appeared negligible, so PCA was used for subsequent processing. Principal components 1 through 10 were utilized, a *k*-nearest neighbor graph was constructed and clustering was performed on the nearest-neighbor graph using a Louvain algorithm and a resolution of 1.0. A Uniform Manifold Approximation and Projection (UMAP) was created for visualization purposes. UMAP projections were constructed using Seurat’s DimPlot function; gene expression patterns were visualized with the FeaturePlot function, and changes in cell percentages and expression levels were displayed using the DotPlot function. For cluster classification, the top expressed genes were identified for each cluster using Seurat’s FindMarkers function using a Wilcoxon rank-sum test.

### Integration of baseline and atherosclerosis Flex scRNA-seq

The processed objects for cKO and iKO studies were read into R and split by condition to create four seurat objects. These four objects underwent a reciprocal PCA (RPCA) integration following the standard Seurat pipeline. PCA analysis was performed, and the top 50 components were stored in the object. Using the common features across conditions, common anchors were found between the datasets using an RPCA analysis and log normalization method where the reduction was specified as ‘rpca’. The data were then integrated together, and the subsequent dataset’s expression was scaled and centered for each gene across all cells while regressing on the mitochondrial percentage, S.Score and G2M.Score. Harmony (version 1.2.0) was run on the integrated data for batch-effect correction, but its effects appeared negligible, so PCA was used for subsequent processing. Principal components 1 through 10 were utilized, a *k*-nearest neighbor graph was constructed, and clustering was performed on the nearest-neighbor graph using a Louvain algorithm and a resolution of 1.0.

### ChIP-seq and analysis

PRDM16 and H3K27ac ChIP-seq were performed on control versus PRDM16-expressing fibroblasts (GSE86017) by Kissig et al.^55^. Target enrichment was calculated as percent input. ChIP-seq reads for PRDM16 and H3K27Ac were aligned to mouse genome mm9 and further processed for peak-calling and genome browser track creation as described^55,56^. For PRDM16 ChIP in aortae, the aortae isolated from 12 mice were dual-crosslinked with 1.5 mM ethylene glycol bis(succinimidyl succinate) for 20 min followed by 1% formaldehyde for 10 min, then snap frozen in liquid nitrogen. Aortae were pooled into a 2-ml safe-lock tube with a 3-mm stainless-steel bead and cryo-milled into a powder using two cycles of 5 Hz for 30 s and 23 Hz for 30 s. Pulverized aortae were then resuspended in 0.6% sodium dodecyl sulfate (SDS) lysis buffer. Chromatin was sheared using a microtip probe sonicator at 25% Amplitude, 30 s on, 20 s off for 5 min. After the first minute of shearing, the insoluble ECM clump was removed with a pipette tip, and shearing resumed for the remaining 4 min. The chromatin preparation was diluted to 0.1% SDS and split into three separate immunoprecipitations (IPs) with ~10 μg chromatin per IP. Samples were incubated with antibodies (Supplementary Table 1) at 4 °C overnight, and Protein A/G magnetic beads were used to capture target chromatin for subsequent washing and elution. DNA was reverse crosslinked overnight at 65 °C with RNase A and treated with proteinase K for 2 h at 50 °C. ChIP DNA was purified using Active Motif Chromatin IP Purification Kit and used for downstream library preparation. Sequencing libraries were made using the Roche KAPA HyperPrep Kit and Roche Universal Dual-Index Adapters. Size selection was performed using Beckman Coulter SPRI Select beads with an 800-bp upper limit cutoff.

For analysis, Fastq files were trimmed using the package trim_galore (version 0.6.10) with default settings and the ‘--paired’ parameter selected. Paired trimmed reads were aligned to the mouse genome (mm10) using Bowtie2 with the parameter ‘-N 1’, and the resulting SAM output was piped into Samtools view (version 1.6) using the parameters ‘-bSF4’. This BAM file was then sorted, duplicate reads were removed, reads that could not be uniquely mapped were dropped, and the file was indexed. The processed BAM files were converted to bigwig files using bamCompare from deeptools (version 3.2.2) comparing with the immunoglobulin G (IgG) sample with the specifications of ‘--binSize 20 --smoothLength 60 --extendReads 150 --effectiveGenomeSize 2653783500 --operation subtract’ for the H3K27ac sample. The same parameters were run for the PRDM16 sample with added specifications of ‘-centerReads’ selected. HOMER (version 4.11) was used for peak calling where tag directories were first created for each sample utilizing makeTagDirectory with the parameters ‘-single -genome mm10’ specified. The function findpeaks was used for peak calling with parameters ‘-size 200 -F 2 -L 2 -fdr 0.01 -center -region’ specified for PRDM16 compared with the IgG input tag directory. The same parameters were run for IgG without calling ‘-i -center’ and ‘-region’ and the H3K27ac sample was run with ‘-style histone’. To identify motifs, Homer’s findMotifsGenome.pl function was run with parameters ‘-dumpFasta -mask’. Homer’s annotatePeaks.pl was used to annotate the called peaks. The fasta output from Homer, along with the motifs, was utilized in MEME Suite (version 5.5.7) in programs tomtom and DREME, which were run with default settings. IGViewer was used to visualize tracks.

### Analysis of human bulk RNA-seq and scRNA-seq

For bulk RNA-seq analysis of early and advanced lesions, human carotid plaques of 38 patients (10 female, 28 male) were prepared and sequenced, and the paired analysis was performed as described by Fidler et al.^27^. The scRNA-seq analysis on 21 coronary endarterectomy samples was performed as described by Bashore et al.^28^. Analysis, quality control and visualization of the feature plots were performed using the Scanpy package in Python.

### Reanalysis of snATACseq dataset

The raw fastq files from Turner et al. (GSE175621)^16^ were acquired using the sratoolkit with the prefetch and fasterq-dump commands. The data were processed using Cell Ranger ATAC (version 7.2.0) with the GRCh38 reference genome and default parameters. The Cell Ranger files were processed in R utilizing Seurat and Signac (version 1.10.0). A unified peak set for all samples was created utilizing the granges function from the GenomicRanges package (version 1.52.0). This unified peak set underwent an interrange transformation using the reduce function from Signac, was filtered to exclude nonstandard chromosomes with coarse pruning mode, blacklisted regions in hg38 were removed, and peaks with widths smaller than 20 bp or larger than 10,000 bp were discarded. After creating the unified peak set among all samples, a matrix of peaks per cell, the chromatin assay object and Seurat object for each sample were made using the commands FeatureMatrix, CreateChromatinAssay and CreateSeuratObject. The single-nucleus (sn)ATAC-seq reads from different individuals were combined at the single-nucleus level, and nuclei were filtered out that had a transcription start site enrichment <0.5 and >6, total number of fragment counts per cell >60,000, percent reads in peak <1, nucleosome signal >2 and a total number of fragments in peaks >30. We then computed the term-frequency inverse-document-frequency on the combined dataset with default parameters and found the top features with a lower percentile bound of ‘q0’. A singular value decomposition was applied to the data. The dimensionality reduction was conducted using a latent semantic indexing algorithm, omitting the first principal component as this component captures sequencing depth rather than biological variation. Therefore, dimensions 2 through 15 were used for cell clustering. Cell clusters were identified by a shared nearest neighbor modularity optimization-based clustering algorithm from Seurat. The smart local moving algorithm was applied for its enhanced efficiency of community detection in large networks with a resolution of 0.1.

Integration of the snATAC-seq dataset (Turner et al.^16^) with the scRNA-seq dataset (Wirka et al.^32^) was performed using the FindTransferAnchors and TransferData functions in the Seurat package. Before the anchor transferring steps, genes in the scRNA-seq mouse dataset were converted to their orthologous versions in common with humans. This was done using the package biomaRt (version 2.56.1) and utilizing the GRCm39 mouse genes and their human ortholog equivalents. A canonical correlation analysis was used for the reduction in finding transfer anchors, dimensions 1 through 50, and the top 2,000 variable features in the reference dataset were used. Afterward, the data were transferred between the two datasets using dimensions 2 through 50, latent semantic indexing (LSI) reduction, a k.weight of 10 and the annotated cell markers established in the single-cell dataset to label the single-nucleus data. The peak expression plots were generated from the Signac package using the CoveragePlot function with window size of 250. The genome track was generated using the package Gviz (version 1.44.1) using the hg38 genome. The vlnplots to show gene expression levels for each cell type were created using the dittoPlot function from the dittoSeq package (version 1.12.1).

### Immunoblotting

Cell lysates were prepared in RIPA buffer (150 mM NaCl, 1% NP-40, 0.1% sodium deoxycholate, 0.1% SDS and 100 mM Tris–HCl, pH 7.4) supplemented with protease inhibitors (Roche) and phenylmethylsulfonyl fluoride (Sigma). Lysates were sonicated, and 4× NuPAGE LDS Sample Buffer (Thermo Scientific) with 10% β-mercaptoethanol was added. Samples were denatured at 95 °C for 5 min, separated on NuPAGE Novex 4–12% Bis-Tris gels (Thermo Scientific) and transferred to Amersham Protran nitrocellulose membranes (Sigma). Membranes were probed with primary antibodies (Supplementary Table 1), followed by detection with horseradish peroxidase-conjugated secondary antibodies (Cell Signaling Technologies) and SuperSignal West Pico PLUS Chemiluminescent Substrate (Thermo Scientific).

### Statistical analysis

For comparisons between two groups, a two-sample *t*-test was used. Comparisons among multiple groups with single or two factors were assessed using one- or two-way analysis of variance (ANOVA), respectively, when passing a Kolmogorov–Smirnov test for normality. When the requirements for normal distribution were not met, samples were analyzed using a Kruskall–Wallis test. To account for multiple comparisons, the Holm–Šídák correction was applied where appropriate. Before analysis, the ROUT (robust regression and outlier removal) method was used to identify and remove outliers. Wound closure rates were fit using a simple linear regression analysis, and an *F*-test for regression analysis was used to assess significance. All data points represent individual, not repeated, measurements.

### Reporting summary

Further information on research design is available in the Nature Portfolio Reporting Summary linked to this article.

## Supplementary information

Reporting Summary

Supplementary Tables Supplementary Tables 1 and 2.

## Source data

Statistical source data for Fig. 1.

Source Data Fig. 2 Statistical source data for Fig. 2.

Source Data Fig. 3 Statistical source data for Fig. 3.

Source Data Fig. 4 Statistical source data for Fig. 4.

Source Data Fig. 6 Statistical source data for Fig. 6.

Source Data Extended Data Fig. 3 Statistical source data for Extended Data Fig. 3.

Supplementary Data 1 Statistical source data for Extended Data Fig. 4.

Source Data Extended Data Fig. 5 Statistical source data for Extended Data Fig. 5.

Source Data Extended Data Fig. 6 Unprocessed western blots.

## Figures and Tables

**Extended Data Table 1:**
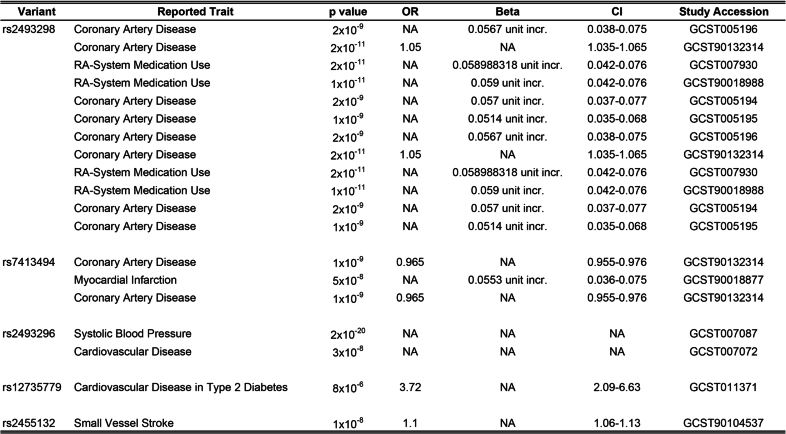
GWAS hits for *PRDM16* related to Coronary Artery Disease. Source: UK biobank

## Data Availability

Sequence datasets are available via GEO with the following accession numbers: ChIP-seq from mouse aortae (GSE305272); bulk RNA-seq of control and *Prdm16* KO mouse aortae (GSE305275) and scRNA-seq of control and *Prdm16* KO mouse aortae under basal and atherogenic conditions (GSE305277). The human coronary artery snATAC-seq data from Turner et al.^16^ are available through GSE175621. The human coronary artery scRNA-seq data from Bashore et al. (Fig. 1) are available through GSE253904 ref. ^28^. The human coronary artery scRNA-seq data (Extended Data Fig. 1) are described in Paloschi et al.^29^ and are available through GSE247238. Bulk RNA-seq of early- and late-stage lesions by Fidler et al.^27^ is available through GSE248395. ChIP-seq data from Kissig et al.^55^ are available through GSE86017. Raw image data are available via figshare at https://figshare.com/s/38dbffa9ed66791f692c (ref. ^57^).

## Code availability

The data were processed using published software tools, for which detailed parameters are described in the Methods. Code is available via GitHub at https://github.com/calhounr/Prdm16-controls-smooth-muscle-cell-fate-in-atherosclerosis.
