# Supplementary material for: PRDM16 regulates smooth muscle cell identity and atherosclerotic plaque composition
Source: Nat Cardiovasc Res. 2025 Oct 17;4(11):1573–88. doi: 10.1038/s44161-025-00737-8 (PMC12611775; doi:10.1038/s44161-025-00737-8)
Supplement: Supplementary file 1 — Reporting Summary [file 44161_2025_737_MOESM1_ESM.pdf]

nature portfolio

Corresponding author(s): Patrick Seale

Last updated by author(s): 09/18/2025

Reporting Summary

Nature Portfolio wishes to improve the reproducibility of the work that we publish. This form provides structure for consistency and transparency in reporting. For further information on Nature Portfolio policies, see our Editorial Policies and the Editorial Policy Checklist.

Statistics

For all statistical analyses, confirm that the following items are present in the figure legend, table legend, main text, or Methods section.

n/a	Confirmed	
☐	☒	The exact sample size (*n*) for each experimental group/condition, given as a discrete number and unit of measurement
☐	☒	A statement on whether measurements were taken from distinct samples or whether the same sample was measured repeatedly
☐	☒	The statistical test(s) used AND whether they are one- or two-sided<br>*Only common tests should be described solely by name; describe more complex techniques in the Methods section.*
☐	☒	A description of all covariates tested
☐	☒	A description of any assumptions or corrections, such as tests of normality and adjustment for multiple comparisons
☐	☒	A full description of the statistical parameters including central tendency (e.g. means) or other basic estimates (e.g. regression coefficient) AND variation (e.g. standard deviation) or associated estimates of uncertainty (e.g. confidence intervals)
☒	☐	For null hypothesis testing, the test statistic (e.g. *F*, *t*, *r*) with confidence intervals, effect sizes, degrees of freedom and *P* value noted<br>*Give P values as exact values whenever suitable.*
☒	☐	For Bayesian analysis, information on the choice of priors and Markov chain Monte Carlo settings
☒	☐	For hierarchical and complex designs, identification of the appropriate level for tests and full reporting of outcomes
☒	☐	Estimates of effect sizes (e.g. Cohen's *d*, Pearson's *r*), indicating how they were calculated

Our web collection on statistics for biologists contains articles on many of the points above.

Software and code

Policy information about availability of computer code

Data collection

Bulk RNAseq: RNA was extracted using TriZol (Invitrogen), and next-generation sequencing (NGS) was performed by Genewiz. The samples were sequenced on the NovaSeq 6000 platform with a paired-end configuration (150 bp). scRNAseq data collection: Fixed aortae were processed using the 10x Flex fRNAseq method. Cell capture, library prep and sequencing was performed by the CHOP single cell technology core. Imaging: Brightfield images were acquired on a Keyence inverted microscope (BZX-710), and fluorescence images were acquired on a Leica SP8x or Leica Stellaris confocal microscope.

Data analysis

qPCR: Real-time qPCR was performed on an ABI7900HT PCR machine using SYBR green fluorescent dye (Applied Biosystems). Fold changes were calculated iusing the delta-delta Ct method, with Tata Binding Protein (Tbp) mRNA serving as a normalization control. Statistical tests and data visualization were performed in GraphPad Prism Version 9.

Bulk RNAseq: Paired-end reads were mapped to Mus musculus reference transcriptome GRCm39 using the command kallisto quant in the Kallisto program (version 0.44.0). The abundance.tsv files generated from kallisto were read into R (version 4.3.0) in R studio (version 3.1.446) using tximport (version 1.28.0). The package AnnotationHub (version 3.8.0) was used for transcript to gene conversion using the available entry "AH98078" for the Ensembl GRCm39 genome build. The R package edgeR (version 3.42.4) was utilized to convert the raw counts to their filtered, library scaled, log Counts Per Million (CPM) with a Trimmed Mean of the M-values (TMM) normalization equivalents. The limma (version 3.56.2) package was used for differential expression analysis using a robust method for fitting the linear model and for computing the empirical bayes statistics. Genes with an adjusted p-value less than 0.05 were considered significant. The volcano and scatter plots were generated using the R package ggplot2 (version 3.4.3). Heatmaps were generated using the R packages pheatmap (version 1.0.12) and complexheatmap (version 2.16.0) for the overlay of bulk and single cell RNA sequencing data. Functional enrichment analysis was performed using the gProfiler2 package (version 0.2.2) and gene set enrichment analysis was done with the fgsea package (version 1.26.0). KO verification was performed with IGViewer by verifying that KO samples had no reads over exon 9 (the floxed exon).

scRNAseq: Fastq files were aligned to the mouse genome (mm10) with cellranger (version 7.2.0) using the command cellranger multi which was aligned to a customized chromium mouse transcriptome probe set (version 1.0.1) to include manually designed probes for Prdm16 Exon 9 detection. The raw matrix files for the Control and Prdm16 Exon 9 Knockout were read into R (version 4.3.0) and processed with DropletUtils (version 1.20.0) where droplets with a false discovery rate less than or equal to 0.01 were retained. These new filtered datasets were then processed with SoupX (version 1.6.2) using it's autoEstCont and adjustCount features to estimate the contamination fraction and then adjust the expression matrices. Taking the newly created ambient RNA expression corrected matrices, we used seurat (version 4.3.0) for quality control processing, filtering, and integration. The function CreateSeuratObject with the parameters "min.features = 20, min.cells = 100" was used to make seurat objects. Then, light filtering of the datasets were performed with the following parameters "nCount_RNA > 500 & nCount_RNA < 20000 & nFeature_RNA > 150 & nFeature_RNA < 8000 & percent.mt < 10". Then samples counts were scaled for each cell by the total number of molecules detected and then multiplied by 10000 and log transformed. The top 2000 most variable features were found in each dataset with a "vst" selection method and the data had its expression scaled and centered for each gene across all cells while regressing on the mitochondrial percentage. Principal component analysis (PCA) was performed where dimensions 1 through 15 were utilized for both datasets as encompassing a majority of the variation in the data. Using these dimensions, a k-nearest neighbor graph was constructed and clustering was performed on the nearest-neighbor graph using a Louvain algorithm and a resolution of 0.2. A uniform manifold approximation and projection (UMAP) was created for visualization purposes. The two processed seurat objects were run through scDblFinder (version 2.0.3) where cells were identified as singlets or doublets. After identifying all cells that were possible doublets, we re-created the starting seurat objects with the same parameters, retained all cells that were identified as singlets, and then re-filtered the data with stricter quality control parameters of "nCount_RNA > 500 & nCount_RNA < 10000 & nFeature_RNA > 500 & percent.mt <= 1" for both datasets. Then, samples counts were scaled for each cell by the total number of molecules detected and then multiplied by 10000 and log transformed. The top 2000 most variable features were found in each dataset with a "vst" selection method. The top 2000 variable genes shared across datasets were selected and common reference points were identified between datasets using a canonical correlation analysis (CCA) and log normalization method. The data was then integrated together and the subsequent dataset's expression was scaled and centered for each gene across all cells while regressing on the mitochondrial percentage, S.Score, and G2M.Score calculated from cell cycle scoring. Harmony (version 1.2.0) was run on the integrated data for batch-effect correction but it's effects appeared neglible so the PCA was utilized for subsequent processing. Principal components 1 through 10 were utilized, a k-nearest neighbor graph was constructed and clustering was performed on the nearest-neighbor graph using a Louvain algorithm and a resolution of 1.0. A UMAP was created for visualization purposes and UMAP projections were constructed using seurat's DimPlot function, gene expression patterns were plotted using FeaturePlot function, and changes in percentage and expression levels in cells using DotPlot. For cluster classification, the top expressed genes were identified for each cluster using seurat's FindMarkers function using a Wilcoxon Rank Sum test and these genes were cross-referenced against the literature and online databases to annotate clusters.

For manuscripts utilizing custom algorithms or software that are central to the research but not yet described in published literature, software must be made available to editors and reviewers. We strongly encourage code deposition in a community repository (e.g. GitHub). See the Nature Portfolio guidelines for submitting code & software for further information.

Data

Policy information about availability of data

All manuscripts must include a data availability statement. This statement should provide the following information, where applicable:

- Accession codes, unique identifiers, or web links for publicly available datasets
- A description of any restrictions on data availability
- For clinical datasets or third party data, please ensure that the statement adheres to our policy

Sequence datasets are available through GEO with the following accession numbers: ChIPseq from mouse aortae (GSE305272); Bulk RNAseq of control and Prdm16 KO mouse aortae (GSE305275) and scRNAseq of control and Prdm16 KO mouse aortae under basal and atherogenic conditions (GSE305277). The human coronary artery snATACseq data from Turner et al.16 is available through GSE175621. The human coronary artery scRNAseq from Bashore et al. (Fig 1) is available through GSE25390428. The human coronary artery scRNAseq (Extended Data Fig. 1) is described in Paloschi et al.29 and is available through GSE247238. Bulk RNAseq of early and late-stage lesions by Fidler et al.27 is available through GSE248395. ChIPseq data from Kissig et al.55 is available through GSE86017. Raw image data is available through https://figshare.com/s/38dbffa9ed66791f692c.

Research involving human participants, their data, or biological material

Policy information about studies with human participants or human data. See also policy information about sex, gender (identity/presentation), and sexual orientation and race, ethnicity and racism.

Reporting on sex and gender	No human studies were used for this study
Reporting on race, ethnicity, or other socially relevant groupings	*Please specify the socially constructed or socially relevant categorization variable(s) used in your manuscript and explain why they were used. Please note that such variables should not be used as proxies for other socially constructed/relevant variables (for example, race or ethnicity should not be used as a proxy for socioeconomic status).*<br>*Provide clear definitions of the relevant terms used, how they were provided (by the participants/respondents, the researchers, or third parties), and the method(s) used to classify people into the different categories (e.g. self-report, census or administrative data, social media data, etc.)*<br>*Please provide details about how you controlled for confounding variables in your analyses.*
Population characteristics	*Describe the covariate-relevant population characteristics of the human research participants (e.g. age, genotypic information, past and current diagnosis and treatment categories). If you filled out the behavioural & social sciences study design questions and have nothing to add here, write "See above."*
Recruitment	*Describe how participants were recruited. Outline any potential self-selection bias or other biases that may be present and how these are likely to impact results.*

Ethics oversight | *Identify the organization(s) that approved the study protocol.*

Note that full information on the approval of the study protocol must also be provided in the manuscript.

Field-specific reporting

Please select the one below that is the best fit for your research. If you are not sure, read the appropriate sections before making your selection.

☒ Life sciences ☐ Behavioural & social sciences ☐ Ecological, evolutionary & environmental sciences

For a reference copy of the document with all sections, see nature.com/documents/nr-reporting-summary-flat.pdf

Life sciences study design

All studies must disclose on these points even when the disclosure is negative.

Sample size: Sample sizes for in vivo atherosclerosis studies were selected based on similar study designs used in the field. In vitro experiments were performed with sample sizes of at least n=3 biological replicates.

Data exclusions: For tail cuff blood pressure measurements, mice were excluded from the analyses if we did not obtain at least 3 consistent measurements. For qPCR gene expression, the ROUT method was used to exclude outliers.

Replication: Replicates were performed as indicated in the text and figure legends for in vitro and in vivo experiments.

Randomization: All animal groups received the same treatment (AAV-PCSK9) so no randomization was performed. Cre+ and Cre- animals were randomly assigned to cages to account for cage effects.

Blinding: During the course of the in vivo studies, the processing of the tissues, atherosclerosis lesion quantification, as well as quantification of all stainings, the investigator was blinded for genotype and study group.

Reporting for specific materials, systems and methods

We require information from authors about some types of materials, experimental systems and methods used in many studies. Here, indicate whether each material, system or method listed is relevant to your study. If you are not sure if a list item applies to your research, read the appropriate section before selecting a response.

Materials & experimental systems

n/a	Involved in the study	
☐	☒	Antibodies
☐	☒	Eukaryotic cell lines
☒	☐	Palaeontology and archaeology
☐	☒	Animals and other organisms
☒	☐	Clinical data
☒	☐	Dual use research of concern
☒	☐	Plants

Methods

n/a	Involved in the study	
☒	☐	ChIP-seq
☒	☐	Flow cytometry
☒	☐	MRI-based neuroimaging

Antibodies

Antibodies used:

Immunostaining:
ACTA2 1:1000 Sigma A5228
TAGLN 1:500 Abcam Ab10135
DAPI 1:1000 Sigma D5942
Ki67 1:250 Abcam ab16667
Lumican 1:200 Novus NBP2-76847
MYH11 1:400 Abcam ab224804
CD68 1:200 Abcam ab955
PRDM16 1:500 Made in house
Perilipin 2 1:200 Novus NB110-40877
PECAM-1 1:200 HistoBioTec DIA-310

Western blotting:
PRDM16 1:500 Made in house
pSmad3 1:1000 Cell Signaling Technologies #9520
Smad3 1:1000 Cell Signaling Technologies #9523
Actin 1:1000 Milipore MAB1501

Validation	Antibodies were validated based on information provided by the vendor, appropriate expression pattern based on changes in gene expression, as well as previous studies. PRDM16 antibody was verified by performing staining or western blotting in KO samples. (p)Smad3 antibody was verified using TGFb treatment.

Eukaryotic cell lines

Policy information about cell lines and Sex and Gender in Research

Cell line source(s)	Primary human aortic smooth muscle cells (hAoSMC) were purchased from Lonza<br>primary human coronary artery SMCs were provided by the Miller lab and their generation was described in Wong et al. 2023 Circ Res<br>BMC fibroblasts were isolated from mouse brown adipose tissue and immortalized as described in Harms et al. (2014)
Authentication	Cell lines were validated by expression of marker genes and appropriate response to treatments (e.g. TGFb)
Mycoplasma contamination	All cells tested negative for mycoplasma
Commonly misidentified lines (See ICLAC register)	n.a.

Animals and other research organisms

Policy information about studies involving animals; ARRIVE guidelines recommended for reporting animal research, and Sex and Gender in Research

Laboratory animals	All mice for this study were male of the C57BL6/J background. The Prdm16 floxed mouse line is available from the Jackson Laboratory: B6.129-Prdm16tm1.1Brsp/J, RRID: IMSR_JAX:024992. They were intercrossed with Tagln-Cre (B6.Cg-Tg(Tagln-cre)1Her/J, Strain #:017491, RRID:IMSR_JAX:017491) or Myh11-CreERT2 (B6.FVB-Tg(Myh11-icre/ERT2)1Soff/J, Strain #:019079, RRID:IMSR_JAX:019079) mice. Where indicated, a tdTomato lineage tracer was included (B6.Cg-Gt(ROSA)26Sortm14(CAG-tdTomato)Hze/J, strain #: 007914).<br><br>Animals were raised at room temperature on standard chow (LabDiet, 5010) with a 12h light–dark cycle at room temperature (22°C) unless specified otherwise. For atherosclerosis experiments, mice were placed at thermoneutrality (30°C) at weaning (21 days old) and maintained at thermoneutrality until the end of the experiment. For iKO studies, tamoxifen (Sigma, T5648; stock 20 mg/mL) was injected intraperitoneally at a dose of 100 mg/kg for 4 consecutive days.<br><br>For atherosclerosis studies, 8 week old mice were retro-orbitally injected with AAV8-PCSK9 D377Y (Vector Biolabs) at a dose of 5 x 10 (11) GC/mouse, as described by to Bjørklund et al. The following day, the mice were put on Western Diet composed of 40% fat and 0.15% cholesterol (Research Diets, D12079B) for 12 or 18 weeks.
Wild animals	N/A
Reporting on sex	Only male mice were used. The Myh11CreER construct is encoded on the Y-chromosome, so it is not possible to create female iKO mice using this strain.
Field-collected samples	N/A
Ethics oversight	All experiments were performed according to procedures approved by the University of Pennsylvania Institutional Animal Care and Use Committee (protocol number 805649). Mice were bred and housed under the care of the University Laboratory Animal Resources at the University of Pennsylvania.

Note that full information on the approval of the study protocol must also be provided in the manuscript.

Plants

Seed stocks	N/A
Novel plant genotypes	*Describe the methods by which all novel plant genotypes were produced. This includes those generated by transgenic approaches, gene editing, chemical/radiation-based mutagenesis and hybridization. For transgenic lines, describe the transformation method, the number of independent lines analyzed and the generation upon which experiments were performed. For gene-edited lines, describe the editor used, the endogenous sequence targeted for editing, the targeting guide RNA sequence (if applicable) and how the editor was applied.*
Authentication	*Describe any authentication procedures for each seed stock used or novel genotype generated. Describe any experiments used to assess the effect of a mutation and, where applicable, how potential secondary effects (e.g. second site T-DNA insertions, mosiacism, off-target gene editing) were examined.*
