# Supplementary figures and images for: PRDM16 regulates smooth muscle cell identity and atherosclerotic plaque composition
Source: Nat Cardiovasc Res. 2025 Oct 17;4(11):1573–88. doi: 10.1038/s44161-025-00737-8 (PMC12611775; doi:10.1038/s44161-025-00737-8)

6b

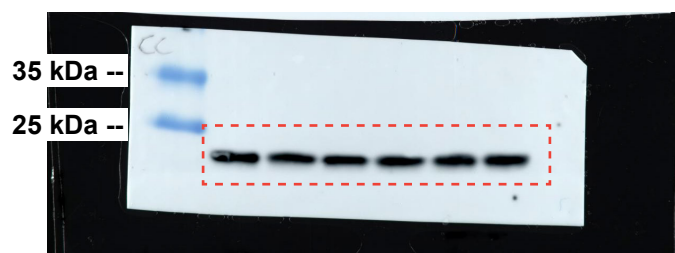

Cofilin

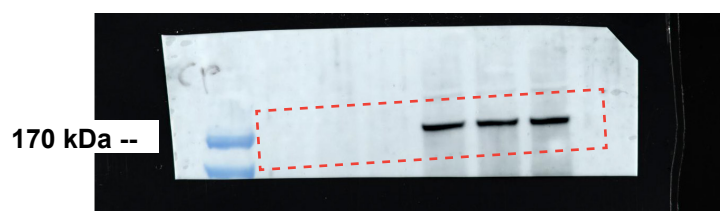

PRDM16

6f

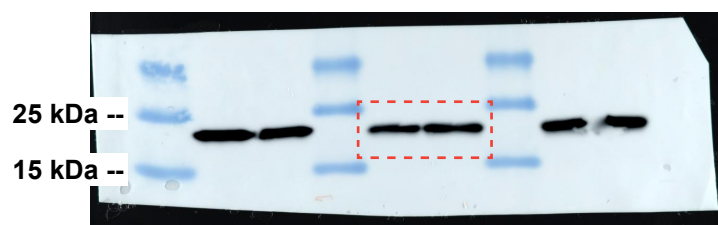

Cofilin

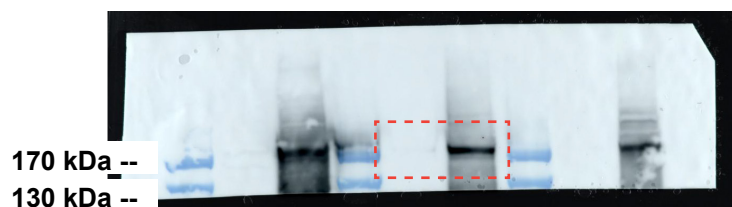

PRDM16

6i

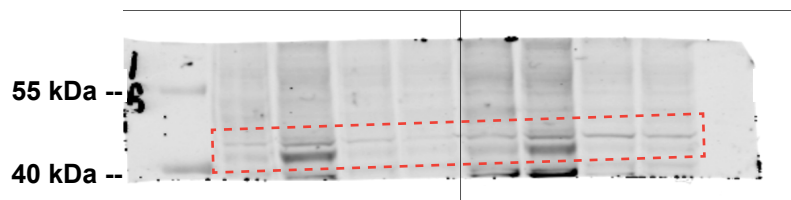

(p)Smad3

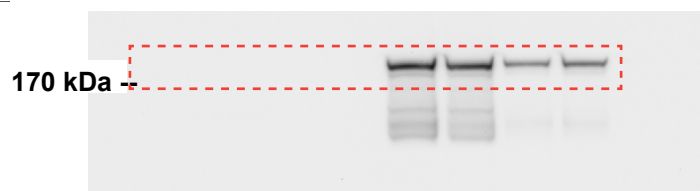

PRDM16

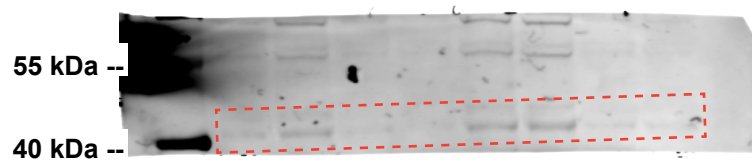

Smad3

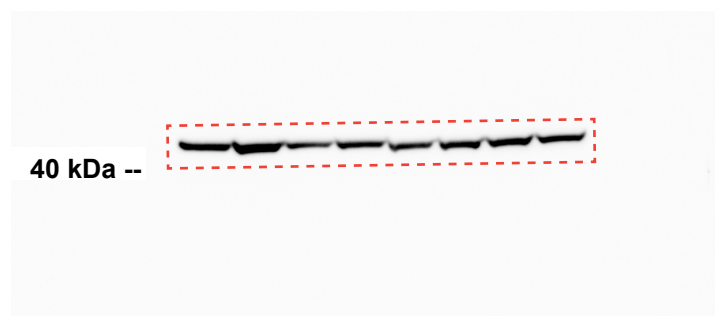

b-Actin

Supplement: Supplementary file 11 — Unprocessed western blots. [file 44161_2025_737_MOESM11_ESM.pdf]
